# Association between expansion of primary healthcare and racial inequalities in mortality amenable to primary care in Brazil: A national longitudinal analysis

**DOI:** 10.1371/journal.pmed.1002306

**Published:** 2017-05-30

**Authors:** Thomas Hone, Davide Rasella, Mauricio L. Barreto, Azeem Majeed, Christopher Millett

**Affiliations:** 1 Public Health Policy Evaluation Unit, Department of Primary Care and Public Health, School of Public Health, Imperial College London, London, United Kingdom; 2 Centre for Data and Knowledge Integration for Health (CIDACS), Instituto Fonçalo Muniz, Fundação Oswaldo Cruz, Salvador, Brazil; 3 Instituto de Saúde Coletiva, Universidade Federal da Bahia, Salvador, Brazil; 4 Center for Epidemiological Studies in Health and Nutrition, University of São Paulo, São Paulo, Brazil; 5 Department of Epidemiology, Institute of Social Medicine, Rio de Janeiro State University, Rio de Janeiro, Brazil; Massachusetts General Hospital, UNITED STATES

## Abstract

**Background:**

Universal health coverage (UHC) can play an important role in achieving Sustainable Development Goal (SDG) 10, which addresses reducing inequalities, but little supporting evidence is available from low- and middle-income countries. Brazil’s Estratégia de Saúde da Família (ESF) (family health strategy) is a community-based primary healthcare (PHC) programme that has been expanding since the 1990s and is the main platform for delivering UHC in the country. We evaluated whether expansion of the ESF was associated with differential reductions in mortality amenable to PHC between racial groups.

**Methods and findings:**

Municipality-level longitudinal fixed-effects panel regressions were used to examine associations between ESF coverage and mortality from ambulatory-care-sensitive conditions (ACSCs) in black/*pardo* (mixed race) and white individuals over the period 2000–2013. Models were adjusted for socio-economic development and wider health system variables. Over the period 2000–2013, there were 281,877 and 318,030 ACSC deaths (after age standardisation) in the black/*pardo* and white groups, respectively, in the 1,622 municipalities studied. Age-standardised ACSC mortality fell from 93.3 to 57.9 per 100,000 population in the black/*pardo* group and from 75.7 to 49.2 per 100,000 population in the white group. ESF expansion (from 0% to 100%) was associated with a 15.4% (rate ratio [RR]: 0.846; 95% CI: 0.796–0.899) reduction in ACSC mortality in the black/*pardo* group compared with a 6.8% (RR: 0.932; 95% CI: 0.892–0.974) reduction in the white group (coefficients significantly different, *p* = 0.012). These differential benefits were driven by greater reductions in mortality from infectious diseases, nutritional deficiencies and anaemia, diabetes, and cardiovascular disease in the black/*pardo* group. Although the analysis is ecological, sensitivity analyses suggest that over 30% of black/*pardo* deaths would have to be incorrectly coded for the results to be invalid. This study is limited by the use of municipal-aggregate data, which precludes individual-level inference. Omitted variable bias, where factors associated with ESF expansion are also associated with changes in mortality rates, may have influenced our findings, although sensitivity analyses show the robustness of the findings to pre-ESF trends and the inclusion of other municipal-level factors that could be associated with coverage.

**Conclusions:**

PHC expansion is associated with reductions in racial group inequalities in mortality in Brazil. These findings highlight the importance of investment in PHC to achieve the SDGs aimed at improving health and reducing inequalities.

## Abstract 2

In a national longitudinal study, Thomas Hone and colleagues show that the expansion of primary healthcare in Brazil was associated with improved health outcomes and reductions in health inequalities between racial groups.

## Author summary

**Why was this study done?:**

- The Sustainable Development Goals include reducing inequalities and making commitments to universal health coverage (UHC).
- There is little evidence about the relationship between expanding primary healthcare (PHC)—as part of the commitment to UHC—and health inequalities, including racial inequalities. This is particularly true for low- and middle-income countries.
- Racial health inequalities are important inequalities to study given the sharp disparities that exist in Brazil and globally.

**What did the researchers do and find?:**

- We examined trends in mortality from ambulatory-care-sensitive conditions for black/*pardo* (mixed race) and white Brazilians from 2000 to 2013, and evaluated whether there were changes in mortality associated with expansion of PHC in municipalities.
- PHC expansion was associated with reductions in mortality for both racial groups, but black/*pardo* Brazilians experienced a 2-fold greater reduction in mortality than white Brazilians.
- The targeted rollout of PHC in Brazil to poorer and smaller municipalities and the greater unmet needs of black/*pardo* Brazilians at the start of the rollout are likely to explain these findings.

**What do these findings mean?:**

- There is evidence of an association between expanded PHC and reductions in health inequalities in Brazil.
- PHC that is preferentially expanded as part of UHC to more disadvantaged populations has the potential to reduce health inequalities.

## Introduction

Reducing inequalities within and among countries is the tenth goal of the Sustainable Development Goals (SDGs). This goal includes the target to “adopt policies, especially fiscal, wage and social protection policies” that “progressively achieve greater equality” (http://www.un.org/sustainabledevelopment/inequality/). Health systems are essential for social protection and, in addition to their contributions to other SDGs for health, may play a vital role in reducing inequalities [1]. Additionally, promoting equality in access to healthcare is a core principle of universal health coverage (UHC) [2]. Investment in primary healthcare (PHC), as part of efforts to achieve UHC, may be especially important in reducing health inequalities [3–5], but evidence is largely derived from North America and Europe.

Brazil is an important setting for evaluating the relationship of PHC with health inequalities. It is a middle-income country with one of the highest levels of income inequality globally (a Gini coefficient of 52.9 in 2013 [6]) and stark health inequalities across income, education, racial, and socio-economic groups [7–13]. Brazil’s considerable investments in social protection policies over the last two decades include the rollout of conditional cash transfers under the Bolsa Família programme and a commitment to UHC with the expansion of PHC through the Estratégia de Saúde da Família (ESF) (family health strategy) [14,15]. The ESF has rapidly expanded since the mid-1990s to become the largest community-based PHC program in the world [16]. In 2014, it covered ~121.2 million individuals (~62.5% of the population) [17]. Family health teams composed of a family doctor, nurses, and community health workers deliver a broad range of comprehensive and preventive healthcare services to defined local populations (approximately 3,400 individuals) [15]. Municipal governments are responsible for the provision of local ESF services, and financial incentives provided by the federal government encourage municipalities to adopt the ESF [18]. In general, municipalities with smaller populations, higher levels of poverty, and a higher proportion of residents from black/*pardo* (mixed race) racial groups exhibited greater uptake of the ESF (S1 Appendix, Figs. A–C) [19]. Expansion of the ESF has been associated with reductions in infant mortality [20–22], deaths from cardiovascular disease [4], and hospitalisations from ambulatory-care-sensitive conditions (ACSCs) [5], but there is little understanding of the associations between ESF expansion and changes in health inequalities. Recent financial and political crises in Brazil are threatening funding for social protection policies, including UHC [23]. Evidence of an association between the ESF and a reduction of inequalities in health outcomes would provide a strong argument for continued investment and political support.

Assessing racial inequalities is important for evaluating the ESF, given the complex historical, sociological, and political dimensions of race in Brazil [24,25]. In contrast to ancestral and ethnic classifications of race in the US and the UK [13], institutions in Brazil use skin colour. Official classifications are *branco* (white), *preto* (black), *pardo* (brown/mixed), *amarello* (Asian), and indigenous, with white, black, and *pardo* accounting for over 98% of the population. Self-reported classification, whilst reflecting ancestral and cultural roots, also reflects an individual’s perceived social identity [11,13,25]. Three main ancestral roots established the Brazilian population today—indigenous individuals, European colonisers, and African slaves [25]. Today, there is considerable admixture (evidenced by a sizeable *pardo* population), but sharp inequalities between racial groups persist [9–13]. Black and *pardo* populations have higher illiteracy, have lower average incomes, and use healthcare services less [9]. In health outcomes, they have lower life expectancy, are affected more by infectious diseases (including tuberculosis, leprosy, leishmaniasis, and schistosomiasis), and have higher mortality rates from external causes, drug overdoses, and homicides [9].

Few studies have examined the potential role of PHC in reducing health inequalities in low- and middle-income countries. This study seeks to address this important gap by examining associations between ESF coverage and mortality from ACSCs in white and black/*pardo* populations in Brazil. We test the hypothesis that expansion of PHC coverage through the ESF in Brazil is associated with reduced inequalities in mortality between racial groups [26].

## Methods

Longitudinal (panel data) regression models were employed using routinely collected municipal-level data, which have been widely applied to evaluate the ESF previously [4,20,22,27–30]. These models estimated associations between ESF coverage and mortality from ACSCs among black/*pardo* and white populations over time, whilst controlling for other confounding factors. The main analysis was restricted to 1,622 municipalities based on previously assessed quality of vital statistics reporting to reduce bias from under-reporting of deaths [31]. Differences in our analytic approach from previous ESF evaluations were necessary to examine associations of ESF expansion and inequalities in mortality between racial groups. These were agreed before compilation and analysis of the data (which commenced in February 2016), and are set out in detail below. In response to reviewers’ suggestions after initial submission, we explored factors associated with ESF uptake, tested for pre-existing trends, tested for biases from ill-defined death adjustments, explored interactions with Bolsa Família, and conducted sensitivity analyses with alternative model specifications and, for comparison with ACSC mortality, on mortality from accidents.

### Data sources

Data from individual death certificates for the years 2000–2013 were obtained from the Brazilian Ministry of Health DATASUS website [32]. Annual municipal population estimates by race and age group based on census data were obtained from the Instituto Brasileiro de Geografia e Estatística (IBGE) website [33]. Municipal-level covariate data, including illiteracy rate, poverty rate, urbanisation rate, and municipal gross domestic product (GDP), were obtained from the IBGE website [33]. Municipal ESF coverage, Bolsa Família coverage, public healthcare spending, the number of public hospital beds, the number of private hospital beds, and private health insurance coverage were obtained from the DATASUS website [32].

### Variables

The mortality rate from ACSCs was the main outcome variable. ACSC deaths were encoded based on a list published by the Brazilian Ministry of Health (and restricted to those aged under 70 y) and ICD-10 codes reported on death certificates (Table 1) [34]. ACSCs were grouped by cause of death into infectious diseases, nutritional deficiencies and anaemia, chronic obstructive pulmonary disease (COPD) and asthma, cardiovascular disease, diabetes, epilepsy, and gastric ulcers. Redistribution of ill-defined deaths was performed using a published and previously utilised methodology to control for confounding trends from reductions in ill-defined deaths over time (S1 Text) [35].

**Table 1:** Ambulatory-care-sensitive conditions with International Classification of Diseases (ICD-10) codes.

Group	Condition	ICD-10 codes
**Infectious diseases**	**Vaccine-preventable diseases**	
	Tetanus	A33–A35
	Diphtheria	A36
	Whooping cough	A37
	Yellow fever	A95
	Acute hepatitis B	B16
	Measles	B05
	Rubella	B06
	Mumps	B26
	Haemophilus meningitis	G00.0
	Tuberculous meningitis	A17.0
	Miliary tuberculosis	A19
	**Preventable conditions**	
	Tuberculosis	A15–A16, A17.1–A17.9, A18
	Acute rheumatic fever	I00–I02
	Syphilis (early and late)	A51–A53
	Malaria	B50–B54
	Ascariasis	B77
	**Gastrointestinal infections and complications**	
	Intestinal infectious diseases	A00–A09
	Dehydration	E86
	**Infections of the ear, nose, and throat**	
	Otitis media	H66
	Acute upper respiratory infections	J00–J03, J06, J31
	**Bacterial pneumonias**	J13–J14, J15.3–J15.4, J15.8–J15.9, J18.1
	**Infections of the kidney and urinary tract**	
	Nephritis	N10–N12
	Cystitis	N30
	Urethritis and urethral syndrome	N34
	Urinary tract infection	N39.0
	**Diseases of the prenatal period and childbirth**	
	Urinary tract infection during pregnancy	O23
	Congenital syphilis	A50
	Congenital rubella	P35.0
	**Infections of the skin and subcutaneous tissue**	A46, L01–L04, L08
	**Pelvic inflammatory disease**	N70–N73, N75–N76
**Nutritional deficiencies and anaemia**	**Anaemia**	D50
	**Nutritional deficiencies**	
	Malnutrition	E40–E46
	Other nutritional deficiencies	E50–E64
**COPD and asthma**	**Asthma**	J45–J46
	**Diseases of the lower respiratory tract**	
	Bronchitis	J20, J21, J40–J42
	Emphysema	J43
	COPD	J44
	Bronchiectasis	J47
**Cardiovascular disease**	**Hypertension**	I10–I11
	**Angina**	I10
	**Heart failure**	I50, J81
	**Cerebrovascular disease**	I63–I67, I69, G45–G46
**Diabetes**	**Diabetes mellitus**	E10–E14
**Epilepsy**	**Epilepsy**	G40–G41
**Gastric ulcers**	**Gastric ulcers**	K25–K28, K92.0, K92.1, K92.2

Source: A list published by Alfradique et al. [34] and developed with the Brazilian Ministry of Health.

COPD, chronic obstructive pulmonary disease.

Race is recorded on death certificates and as part of the decennial census in Brazil. Census recording of race is self-reported. Individuals select *branco* (white), *preto* (black), *pardo* (brown/mixed), *amarello* (Asian), or indigenous. Recording of race on death certificates (using the same categories) is usually completed by the physician certifying the death and should be based on input from the family [13]. *Amarello* and indigenous deaths were very few and not examined. Black and *pardo* deaths were merged into one group, despite issues regarding differences between these populations [13]. This was to overcome potential differences in racial classification of individuals occurring either between censuses and death certificates, or over time as individuals and/or society changed reporting behaviour. Whilst evidence indicates overlap between black and *pardo* classifications in reporting of race, there are significantly clearer divisions between white and *pardo* classifications [36].

Reporting of race is near complete in censuses (99.29% in 2000 and 99.98% in 2010) and high on death certificates (total missing for 2000–2013 was 5.8%). For completeness, values were imputed for certificates with race missing using other death certificate variables (sex, age, education level, marital status, and location of death) and municipal population estimates of racial groups (S2 Text). For the period 2000–2013, race was imputed for 39,198 of the total 588,872 ACSC deaths (of those white or black/*pardo* and aged under 70 y) in the municipalities included in the analysis.

Using municipal census population data, population distributions by race (white and black/*pardo*) and age group (0–4, 5–9, 10–14, 15–19, 20–24, 25–29, 30–39, 40–49, 50–59, and 60–69 y) were calculated for each municipality for the census years (2000 and 2010), and were linearly interpolated and extrapolated for non-census years (2001–2009 and 2011–2013). Annual total municipal population estimates were used to calculate annual age and race group population estimates for each municipality. Direct age standardisation of cause of death by race was performed, producing annual age-adjusted mortality rates for total ACSCs and ACSC groups by race. The dependent variables (for each municipality and for each year) in the regression models were the expected (from age standardisation) number of deaths from ACSCs (in total and by ACSC group) for the black/*pardo* and white populations and the standardised rate ratio (SRR) between total black/*pardo* and white ACSC mortality rates. Rate ratios (RRs) are commonly used metrics for comparing rates between groups (e.g., between males and females) [37]. In this study, the ACSC mortality rate for the black/*pardo* population was divided by the ACSC mortality rate for white population.

The main variable of interest was municipal ESF coverage (percent) of the population, with official calculations based on one ESF team per 3,450 individuals [17]. A 2-y average (within the year and the year prior) of ESF coverage was employed, even though comparable results were obtained with just within-year coverage or including 2- and 3-y lags. This approach was used to account for varying lagged and duration effects of the ESF that may differ between conditions and populations, to account for the time for ESF services to become fully operational and effective, and to permit simple comparison between the two racial groups.

Annual municipality-level covariate data were selected to include variables relating to socio-economic development, income, and the health system, which have been shown to affect mortality [38,39]. The covariates were scaled as percentages, in hundreds of Brazilian reais (R$100s) per person (adjusted for inflation), or per 1,000 inhabitants. Variables expressed as percentages were scaled between 0 and 1 so a one-unit increase would represent a 100% increase. Where necessary, logarithms were used to improve model fit. Covariates employed in all models were: Bolsa Família coverage (percent), illiteracy rate in those over 25 y (percent) (log-transformed), poverty rate (percent), population living in urban areas (percent), public healthcare spending (R$100s per person), public hospital beds per 1,000 population, private hospital beds per 1,000 population, private healthcare insurance (percent) (log-transformed), and GDP per person (R$100s per person) (log-transformed). An interaction between private healthcare insurance (percent) (log-transformed) and GDP per person (R$100s per person) (log-transformed) was included for model fit.

### Statistical analysis

Descriptive analyses were undertaken, including national trends of ACSC mortality rates for black/*pardo* and white populations and the national SRR of the two rates.

Fixed-effects longitudinal regression was employed as an appropriate method for analysing annual observations of municipalities [40]. Fixed-effects models control for time-invariant unobserved factors that may affect mortality and could bias the results [40]. Consequently, only changes *within* municipalities over time are estimated rather than differences *between* municipalities. We tested for pre-intervention trends (i.e., mortality rates prior to ESF adoption and expansion) to determine whether time-varying unobserved factors could bias the results. Examining trends in the years 2000–2003 (when many municipalities still had relatively low coverage) and employing dummy variables for the years prior to ESF adoption revealed no evidence of pre-intervention trends.

In the models with dependent count variables (ACSC deaths), a Poisson model with a population offset term was employed, allowing the dependent variable (ACSC deaths) to be modelled as a rate (deaths per population). To aid interpretability, the coefficients were exponentiated and reported as RRs. These are interpreted as a ratio of the mortality rates for a one-unit increase in the independent variable (e.g., a 100% increase in ESF coverage or an additional year during the study period) (see S3 Text for more details). In other words, the difference between 1 and the RR can be interpreted as the percentage change in the rate given a one-unit increase in the independent variable. For the SRR, linear longitudinal regression was employed, and β coefficients reported. These are interpreted as the change in the SRR given a one-unit increase (i.e., from 0% to 100% coverage).

Two multiple regression models were undertaken examining the association between ESF expansion and ACSC mortality in the black/*pardo* and white populations separately. Differences in the effect sizes were tested for statistical significance (S4 Text). The *p*-values for the differences between the coefficients from the two models are reported in the text. The association between ESF coverage and the SRR was examined with a multiple regression model. Several regression models for the groups of ACSCs (infectious diseases, nutritional deficiencies and anaemia, COPD and asthma, cardiovascular disease, diabetes, epilepsy, and gastric ulcers) were employed in the black/*pardo* and white populations separately. Small numbers prohibited the use of SRR for groups of ACSCs. In all models, municipality-clustered robust standard errors were employed to account for possible auto-correlation and heteroscedasticity [40]. Stata 12 was used for statistical analysis.

### Sensitivity analyses

Multiple sensitivity analyses were undertaken to check the robustness of the findings. First, alternative model specifications with sequential addition of covariates, random-effect models, and negative-binomial models were employed (S2 Appendix, Tables A and B). Second, varying classifications of ESF coverage were tested (S2 Table). Third, mortality from accidents (ICD-10 V01–X59) was tested, as an outcome that should have no association with ESF expansion (S3 Table). Fourth, the validity of imputing race on death certificates with race missing was assessed by excluding deaths where race was not recorded (S3 Appendix, Tables A and B). Fifth, the validity of redistributing ill-defined causes of death was tested (S4 Appendix, Tables A and B). Sixth, the analyses were repeated using data from all 5,565 municipalities in Brazil, not just those with adequate recording of vital statistics (S5 Appendix, Tables A and B). Seventh, because the potential for misclassification of race on death certificates exists (between the white and black/*pardo* populations), the effect of reclassifying black/*pardo* deaths (which are higher) as white was examined (S6 Appendix, Tables A–H). Eighth, an interaction between Bolsa Família and ESF coverage was examined (S7 Appendix).

## Results

**Fig 1:**
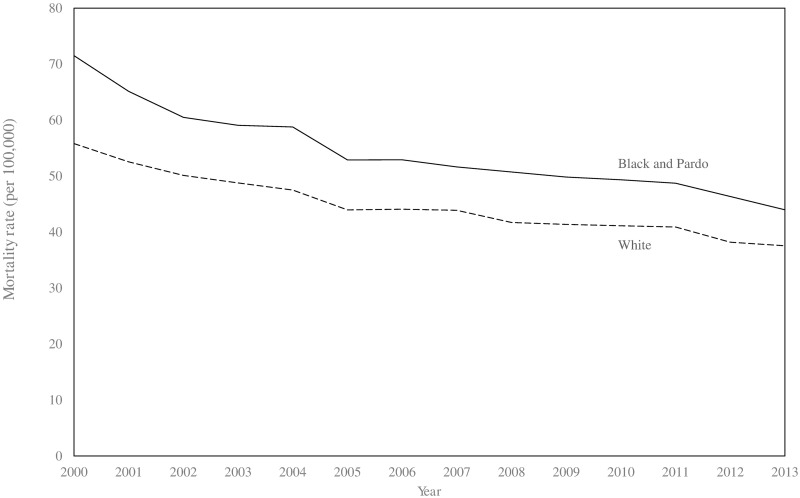
Age-standardised mortality rates for ambulatory-care-sensitive conditions in black/*pardo* and white populations in Brazil (2000–2013). Data only from 1,622 municipalities with adequate reporting of vital statistics. Mortality rates are age standardised to the 2010 national population estimates.

**Fig 2:**
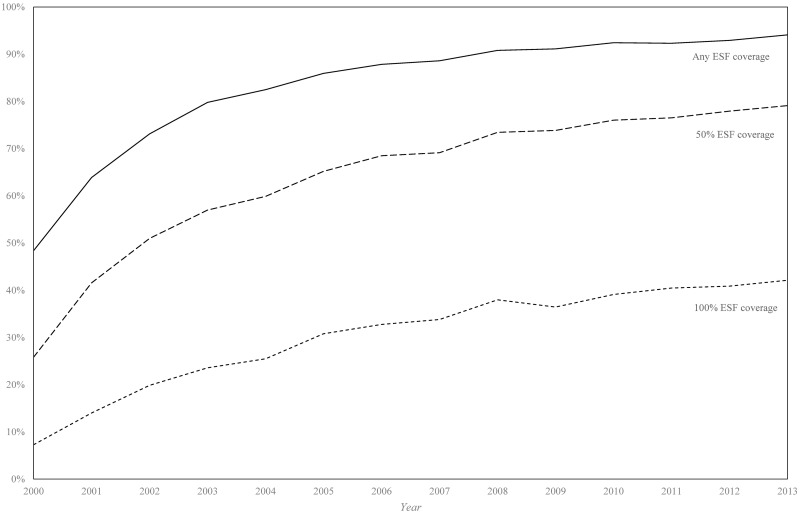
Percentage of municipalities achieving any, 50%, and 100% Estratégia de Saúde da Família coverage (2000–2013). Only data from the 1,622 municipalities with adequate vital statistics reporting are included in this analysis. ESF, Estratégia de Saúde da Família.

Between 2000 and 2013, there were 281,877 and 318,030 deaths from ACSC causes in the black/*pardo* and white populations, respectively (after age standardisation). Age-standardised ACSC mortality rates fell 37.9%, from 93.3 to 57.9 per 100,000, in the black/*pardo* population and by 34.9%, from 75.7 to 49.2 per 100,000, in the white population (Fig 1; S7 Appendix). Mortality from ACSC causes in the black/*pardo* population was between 17% and 23% higher than in the white population during the study period. There was a sizeable expansion of the ESF over the period, both in terms of the number of municipalities adopting the ESF and the average municipal ESF coverage (Fig 2).

In longitudinal Poisson regression models, ACSC mortality decreased annually by 3.4% (RR: 0.966; 95% CI: 0.954–0.976) in the black/*pardo* population and by 2.9% (RR: 0.971; 95% CI: 0.963–0.979) in the white population in adjusted models (Table 2). ESF expansion (from 0% to 100% coverage) was associated with a 15.4% (RR: 0.846; 95% CI: 0.796–0.899) reduction in ACSC mortality in the black/*pardo* population and a 6.8% (RR: 0.932; 95% CI: 0.892–0.974) reduction in the white population. These coefficients were significantly different (*p* = 0.012).

**Table 2:** Results from longitudinal fixed-effects Poisson regression of mortality from ambulatory-care-sensitive conditions in black/*pardo* and white populations.

Variable	Black/*pardo* group		White group	
	RR or *N*	95% CI	RR or *N*	95% CI
ESF coverage	0.846***	0.796, 0.899	0.932**	0.892, 0.974
Year	0.966***	0.954, 0.979	0.971***	0.963, 0.979
Bolsa Família coverage	0.873*	0.784, 0.973	0.895	0.799, 1.002
Illiteracy rate	0.940	0.757, 1.168	0.905	0.796, 1.029
Poverty rate	1.592*	1.053, 2.407	1.149	0.849, 1.555
Urbanisation rate	1.135	0.697, 1.848	0.839	0.585, 1.204
Public healthcare spending	1.009	1.000, 1.019	1.002	0.991, 1.013
Public hospital beds	1.001	0.941, 1.065	1.013	0.960, 1.069
Private hospital beds	1.193	0.913, 1.561	1.088	0.922, 1.284
Private healthcare insurance	0.831**	0.744, 0.928	0.900*	0.827, 0.979
GDP	0.846**	0.759, 0.944	0.853***	0.781, 0.932
Private healthcare insurance × GDP	0.953***	0.934, 0.972	0.974**	0.959, 0.990
*N* observations	22,384		22,694	
*N* municipalities	1,599		1,621	

Exponentiated coefficients:

* *p <* 0.05,

** *p <* 0.01,

*** *p <* 0.001.

The study period was from 2000 to 2013. Robust standard errors were employed. ESF coverage is a 2-y average of within-year municipal ESF coverage and coverage in the year before. Year is a continuous variable and is interpreted as the underlying annual change in mortality rate during the study period. ESF coverage, Bolsa Família coverage, poverty rate, and urbanisation rate are all expressed as percentages and are scaled so a one-unit increase represents a 100% increase. Private healthcare insurance is also expressed as a percentage, but is log-transformed. Illiteracy rate is the illiteracy rate of those aged 25 y and over and is log-transformed. Public healthcare spending is expressed as hundreds of Brazilian reais per person, as is GDP, although GDP is log-transformed. Public and private hospital beds are expressed per 1,000 municipal inhabitants. Some municipalities and/or year observations are not included for a racial group due to there being no deaths from ambulatory-care-sensitive conditions for that racial group.

95% CI, 95% confidence interval; ESF, Estratégia de Saúde da Família; GDP, gross domestic product; RR, rate ratio.

ESF expansion (from 0% to 100% coverage) was associated with a 0.179 reduction (95% CI: 0.022–0.336) in the SRR (Table 3). Predicted SRRs from the model demonstrate that if ESF coverage were 0% in all municipalities, mortality amenable to PHC in the black/*pardo* population would be 29.6% higher than that in the white population (an estimated SRR of 1.296). With 100% ESF coverage in all municipalities, mortality amenable to PHC in the black/*pardo* population would be 11.7% higher than that in the white population (an estimated SRR of 1.117). Thus, expansion of the ESF (from 0% to 100%) yields a 60.5% reduction in the excess mortality that the black/*pardo* population experiences over the white population.

**Table 3:** Results from the longitudinal fixed-effects linear regression of standardised rate ratios for mortality from ambulatory-care-sensitive conditions in black/*pardo* and white populations.

Variable	Coefficient or *N*	95% CI
ESF coverage	−0.179*	−0.336, −0.022
Year	0.010	−0.021, 0.041
Bolsa Família coverage	−0.170	−0.549, 0.209
Illiteracy rate	0.066	−0.476, 0.609
Poverty rate	1.226*	0.167, 2.284
Urbanisation rate	0.847	−0.361, 2.056
Public healthcare spending	0.012	−0.014, 0.039
Public hospital beds	−0.082	−0.217, 0.054
Private hospital beds	0.088	−0.207, 0.382
Private healthcare insurance	−0.114	−0.405, 0.177
GDP	−0.132	−0.450, 0.185
Private healthcare insurance × GDP	−0.038	−0.095, 0.019
*N* observations	21,336	
*N* municipalities	1,622	

* *p <* 0.05.

The study period was from 2000 to 2013. Robust standard errors were employed. ESF coverage is a 2-y average of within-year municipal ESF coverage and coverage in the year before. Year is a continuous variable and is interpreted as the change in mortality rate for each additional year. ESF coverage, Bolsa Família coverage, poverty rate, and urbanisation rate are all expressed as percentages and scaled so a one-unit increase represents a 100% increase. Private healthcare insurance is also expressed as a percentage, but is log-transformed. Illiteracy rate is the illiteracy rate of those aged 25 y and over and is log-transformed. Public healthcare spending is expressed as hundreds of Brazilian reais per person, as is GDP, although GDP is log-transformed. Public and private hospital beds are expressed per 1,000 municipal inhabitants. Some municipalities and/or year observations are not included for a racial group due to there being no deaths from ambulatory-care-sensitive conditions for that racial group.

95% CI, 95% confidence interval; ESF, Estratégia de Saúde da Família; GDP, gross domestic product.

### Associations with cause-specific mortality

Over the study period, mortality from COPD and asthma decreased annually by 4.1% (RR: 0.959; 95% CI: 0.933–0.985) in the black/*pardo* population and by 4.5% (RR: 0.955; 95% CI: 0.939–0.971) in the white population (Table 4). Mortality from cardiovascular disease decreased annually by 3.7% (RR: 0.963; 95% CI: 0.948–0.979) in the black/*pardo* population and by 2.7% (RR: 0.973; 95% CI: 0.962–0.984) in the white population. For the black/*pardo* population, mortality from diabetes decreased 2.7% per year (RR: 0.973; 95% CI: 0.952–0.994), whilst there were non-significant trends in infectious diseases, nutritional deficiencies and anaemia, epilepsy, and gastric ulcers. For the white population, mortality from infectious diseases decreased 2.8% annually (RR: 0.972; 95% CI: 0.948–0.997), mortality from nutritional deficiencies and anaemia decreased 4.9% annually (RR: 0.951; 95% CI: 0.909–0.994), and mortality from gastric ulcers decreased 4.9% annually (RR: 0.951; 95% CI: 0.922–0.981), but there were no significant trends in diabetes and epilepsy mortality.

**Table 4:** Results from longitudinal fixed-effects Poisson regressions for mortality by groups of ambulatory-care-sensitive conditions in black/*pardo* and white populations.

Group	Variable	Black/*pardo* group		White group	
		RR or *N*	95% CI	RR or *N*	95% CI
**Infectious diseases**	ESF coverage	0.725***	0.620, 0.848	0.956	0.854, 1.070
	Year	0.989	0.953, 1.026	0.972*	0.948, 0.997
	*N* observations	18,046		21,476	
	Total deaths	35,353		31,716	
**Nutritional deficiencies and anaemia**	ESF coverage	0.721**	0.578, 0.899	1.251*	1.011, 1.548
	Year	0.982	0.937, 1.031	0.951*	0.909, 0.994
	*N* observations	11,662		15,932	
	Total deaths	5,988		5,313	
**COPD and asthma**	ESF coverage	1.072	0.939, 1.223	0.988	0.914, 1.068
	Year	0.959**	0.933, 0.985	0.955***	0.939, 0.971
	*N* observations	19,880		22,120	
	Total deaths	27,174		48,055	
**Cardiovascular**	ESF coverage	0.871**	0.801, 0.947	0.929*	0.876, 0.985
	Year	0.963***	0.948, 0.979	0.973***	0.962, 0.984
	*N* observations	21,853		22,652	
	Total deaths	137,061		147,682	
**Diabetes**	ESF coverage	0.807***	0.713, 0.912	0.932	0.849, 1.023
	Year	0.973*	0.952, 0.994	0.987	0.971, 1.004
	*N* observations	20,244		22,526	
	Total deaths	54,873		65,003	
**Epilepsy**	ESF coverage	0.961	0.745, 1.240	1.017	0.806, 1.284
	Year	1.000	0.949, 1.054	0.962	0.921, 1.005
	*N* observations	11,578		15,848	
	Total deaths	4,045		4,908	
**Gastric ulcers**	ESF coverage	0.962	0.915, 1.012	0.951**	0.922, 0.981
	Year	0.884	0.697, 1.122	0.939	0.800, 1.103
	*N* observations	13,230		18,788	
	Total deaths	8,542		10,798	

Exponentiated coefficients:

* *p <* 0.05,

** *p <* 0.01,

*** *p <* 0.001.

The table shows select results from longitudinal Poisson regressions for groups of ambulatory-care-sensitive conditions for both the black/*pardo* population and the white population, in addition to the number of deaths for each group of conditions and racial group. The study period was from 2000 to 2013. Robust standard errors were employed. ESF coverage is a 2-y average of within-year municipal ESF coverage and coverage in the year before, and is expressed as percentages and scaled so a one-unit increase represents a 100% increase. Year is a continuous variable and is interpreted as the change in mortality rate for each additional year. Although not reported, all regressions control for Bolsa Família coverage (percent), illiteracy rate of those over 25 y (log-transformed), poverty rate (percent), urbanisation rate (percent), public healthcare spending (R$100s per person), public hospital beds per 1,000 population, private hospital beds per 1,000 population, private healthcare insurance (percent) (log-transformed), GDP per person (R$100s per person) (log-transformed), and the interaction of private healthcare insurance (percent) (log-transformed) and GDP per person (R$100s per person) (log-transformed). Some municipalities and/or year observations are not included for a racial group due to there being no deaths from ambulatory-care-sensitive conditions for that racial group.

95% CI, 95% confidence interval; COPD, chronic obstructive pulmonary diesease; ESF, Estratégia de Saúde da Família; GDP, gross domestic product; R$100s, hundreds of Brazilian reais per person; RR, rate ratio.

ESF expansion (from 0% to 100%) was associated with a decrease in mortality from cardiovascular disease of 12.9% (RR: 0.871; 95% CI: 0.801–0.947) and 7.1% (RR: 0.929; 95% CI: 0.876–0.985) in the black/*pardo* and white populations, respectively. In the black/*pardo* population, ESF expansion was associated with 27.5% lower mortality from infectious diseases (RR: 0.725; 95% CI: 0.620–0.848) and 19.3% lower mortality from diabetes (RR: 0.807; 95% CI: 0.713–0.912), but there was no significant association with mortality for these ACSC groups in the white population. ESF expansion was associated with 17.9% lower mortality from nutritional deficiencies and anaemia (RR: 0.721; 95% CI: 0.478–0.899) in the black/*pardo* population, but in the white population, it was associated with 25.1% higher mortality (RR: 1.251; 95% CI: 1.011–1.548). For both the black/*pardo* and white populations, there was no significant association between ESF and mortality from COPD and asthma, epilepsy, or gastric ulcers.

### Sensitivity analyses

Sensitivity analyses demonstrate the robustness of our findings. Alternative model specifications (S2 Appendix, Tables A and B) demonstrate the stability and robustness of the findings. We found that controlling for additional factors (fixed effects, covariates, and state-year fixed effects) did not change our findings; in fact, the differential associations of the ESF with black/*pardo* and white mortality became more apparent when these factors were taken into account. Alternative classifications of ESF coverage did not change the overall differences in the associations between ESF expansion and black/*pardo* and white mortality, although the results of the sensitivity analysis did suggest that greater reductions in mortality in the black/*pardo* population accrued over a longer period (S2 Table).

Examining mortality from accidents as a control outcome revealed no significant association of accident deaths with ESF coverage in either racial group, adding to the robustness of our findings (S3 Table). Excluding deaths with race not recorded yielded highly comparable results, demonstrating that imputation of missing race data was not a source of bias (S3 Appendix, Tables A and B). Repeating the analysis without adjustment for ill-defined deaths produced similar results (S4 Appendix, Tables A and B). An analysis with all 5,565 municipalities in Brazil (not just those with adequate reporting of vital statistics) found lower ACSC mortality associated with ESF expansion only in the white population yet a highly comparable association between ESF expansion and changes in the SRR (S5 Appendix, Tables A and B). The non-significance of the association of ACSC mortality with ESF expansion in the black/*pardo* population (when including municipalities with inadequate reporting of vital statistics) is expected given the likelihood of black/*pardo* deaths being under-reported and the role of the ESF in reducing under-reporting [41]. To examine the extent to which misclassification bias (i.e., deaths that were encoded as black/*pardo* when individuals self-identified as white in the census) could affect the results, we randomly reclassified 10%, 20%, and 30% of black/*pardo* deaths as white deaths (S6 Appendix, Tables A–H). A similar association between ESF expansion and race-specific mortality was found even when 30% of black/*pardo* deaths were reclassified, although associated reductions in inequality were lower. Lastly, an interaction between Bolsa Família and ESF coverage was non-significant (S7 Appendix, Tables A–C).

## Discussion

Expansion of the ESF between 2000 and 2013 in Brazil was associated with a 2-fold greater reduction in ACSC mortality in the black/*pardo* compared to the white population. This differential benefit reduced racial inequalities in mortality and was driven by greater reductions in deaths from infectious diseases, nutritional deficiencies and anaemia, diabetes, and cardiovascular disease in the black/*pardo* population. This paper provides further evidence of the importance of expanding UHC in low- and middle-income countries.

Previous literature indicates that ESF expansion is associated with reduced child mortality, mortality from cardiovascular disease, and ACSC hospitalisations [4,5,20–22]. These changes are likely due to improved access to healthcare and a focus on prevention, health promotion, proactive outreach, and early management of conditions within the ESF [3]. Whilst there is local variation in how the ESF is implemented, federal guidelines specify minimum mandatory strategic areas ESF teams must cover, including the management of hypertension, diabetes, tuberculosis, and women’s and children’s health [42]. In this study, ESF expansion was associated with reductions in mortality in the ACSC groups that mirror these mandatory strategic areas. We found that ESF expansion was associated with reductions in cardiovascular mortality of 12.9% and 7.1% in the black/*pardo* and white groups, respectively. We found a 27.5% reduction in mortality from infectious diseases with ESF expansion in the black/*pardo* population. ESF expansion was also associated with a 17.9% reduction in mortality from nutritional deficiencies and anaemia in the black/*pardo* population, with children under 5 y accounting for over 25% of these deaths (compared to roughly 3% of all deaths from ACSCs). Additionally, ESF-associated reductions in mortality from respiratory diseases (COPD and asthma), epilepsy, and gastric ulcers are consistent with their inclusion within ACSC definitions and the fact that these conditions are considered amenable to PHC. We found no association between ESF expansion and mortality from accidents, which is not considered sensitive to primary care, providing reassurance that the associations of ESF expansion with ACSCs reported are not due to confounding.

The differential associations between ESF expansion and mortality in black/*pardo* and white populations may be explained by numerous factors, with socio-economic differences a key explanatory factor. Black/*pardo* populations are disproportionately affected by diseases of poverty, including infectious diseases, malnutrition, and anaemia [9,43], but these conditions may be more responsive to ESF services as they are generally easier to treat in PHC settings than complex non-communicable diseases. Additionally, black/*pardo* populations in Brazil have lower utilisation of healthcare and higher rates of forgone healthcare [9], suggesting ESF expansion may have facilitated access to healthcare and reduced unmet need. Lastly, the finding that ESF has benefitted black/*pardo* populations more than white populations may not be surprising given that ESF expansion had been prioritised within poorer areas and municipalities. Surveys indicate that black/*pardo* populations now have greater ESF coverage (at 57.3% in 2008) than white populations (44.6% in 2008), but lower coverage of private health insurance, suggesting they are more reliant on publicly funded and provided services, including the ESF [9].

Our findings are consistent with evidence derived largely from studies conducted in North America and Europe that show “equity-enhancing” associations from PHC expansion [3]. However, these studies mostly examine associations of PHC with health inequalities across income groups. There are fewer studies examining the association of PHC with health inequalities between racial groups. In a study in the US, increasing the supply of primary care physicians was associated with larger reductions in African-American mortality than white mortality [38]. Inequalities in low birth weight between African-American and white infants are lower among those using PHC [44]. No evidence exists on the association between PHC and race in Brazil, although a few studies have examined inequalities between municipalities. Previous Brazilian studies have shown that ESF expansion was associated with greater reductions in infant mortality in municipalities with higher infant mortality at baseline [20,45]. Another study demonstrated greater reductions in infant mortality in municipalities with lower human development, also implying improvements in equity [20].

There are important limitations to this study pertinent to the interpretation. First, these analyses were conducted on municipal-level aggregated data, and more complete, individual data with ESF enrolment, consultation rates, and associated health outcomes are required to elucidate the mechanisms determining the greater benefits experienced by the black/*pardo* population. Second, there could be biases from the methods employed and data manipulation. However, we conducted extensive sensitivity analyses that showed that our findings are robust to ill-defined death reclassification, varying classifications of ESF coverage, and alternative model specifications. We also found no evidence of pre-intervention trends that would bias the findings. Third, there are important caveats regarding the use of race in this study. There is potential for misclassification bias of race (with race in censuses self-reported and race in death certificates reported by either the family or physician), although sensitivity analyses indicate the robustness of the findings. Black/*pardo* would have to be incorrectly recorded for over 30% of black/*pardo* deaths for the differences found to be non-significant. Additionally, by grouping together black and *pardo* deaths, we do not account for the large amount of heterogeneity in health outcomes between these groups [13]. Fourth, lack of statistical power due to small numbers is apparent in our analysis of associations between ESF expansion and cause-specific deaths. This precluded any potential analysis with SRRs for ACSC groups. Fifth, we used mortality from ACSCs as our outcome measure rather than the more broadly defined concept of healthcare-amenable mortality [30,46]). This was principally to focus on conditions that have been defined as amenable to PHC within the Brazilian context and to exclude those that may be strongly influenced by hospital-based care. While previous research has generally examined hospital admissions for ACSCs, this was not feasible here due to low recording of race in hospital admission data in Brazil. We present a comparison of conditions included in the Brazilian Ministry of Health’s definition of ACSCs and healthcare-amenable mortality as defined by Nolte and McKee [46] in S4 Table.

Policy-makers should note that in Brazil, where sharp inequalities persist and an ambition to achieve UHC has been boldly pursued over the last 20 years, the equity-promoting associations of PHC are evident [30]. The strong positive relationship between PHC and reduced racial inequalities in mortality provides impetus for a renewed government commitment to the ESF. Current proposals that could limit public spending in Brazil and cause disinvestment from social protection programmes, including the ESF [23], may reverse the valuable progress made towards reducing health inequalities in the country. The health inequality impacts of policy changes influencing the ESF, which is the primary vehicle for UHC in Brazil, should be carefully monitored and evaluated.

Beyond the equity-enhancing nature of PHC itself, the impressive reductions in inequality in ACSC deaths between racial groups seen in Brazil may have been facilitated by numerous factors. These include the more rapid expansion of the ESF in poorer and more deprived areas, and the proactive outreach healthcare delivered by community health workers. Whilst challenges exist, including retaining health professionals in rural areas [15] and a lack of coverage for the urban poor [47], there are valuable lessons for other countries from Brazil’s efforts to achieve UHC. The pro-equity health gains demonstrated here reflect the country’s adoption of a pro-poor pathway to UHC. Universal access was embraced from the start, services are publicly financed, there is a focus on expanding access through community-based models of care, and strong political commitment has enabled rapid and sizeable expansion [48]. Valuable lessons may be derived from other settings including Costa Rica, which similarly expanded PHC in poorer areas preferentially [49], and countries such as Tanzania, Uganda, and Chile, which have accelerated coverage in underserved areas through flexible budget allocations [50].

In conclusion, expansion of the ESF in Brazil was associated with improved health outcomes and reductions in health inequalities between racial groups. As countries aim to “progressively achieve greater equality” as part of the SDGs, these findings reinforce the importance of strong PHC-focused health systems for improving health and reducing health inequities.

## Supporting information

S1 Alternative Language Abstract Portuguese translation of the abstract by Leandro Garcia. (DOCX)

S1 Appendix Expansion of the Estratégia de Saúde da Família by municipal population, poverty rate, and black/*pardo* population. (DOCX)

S2 Appendix Sensitivity analysis: Sequential addition of covariates and alternative model specifications. (DOCX)

S3 Appendix Sensitivity analysis: Excluding deaths with missing race. (DOCX)

S4 Appendix Sensitivity analysis: No adjustment for ill-defined deaths. (DOCX)

S5 Appendix Sensitivity analysis: Analysing all municipalities (including those with inadequate vital statistics reporting). (DOCX)

S6 Appendix Sensitivity analysis: Testing potential misclassification bias. (DOCX)

S7 Appendix Sensitivity analysis: Interaction between Bolsa Família and Estratégia de Saúde da Família coverage. (DOCX)

S1 STROBE Checklist STROBE checklist. (DOC)

S1 Table National age-standardised mortality rates (deaths per 100,000) from ambulatory-care-sensitive conditions in black/*pardo* and white groups, absolute difference in rates, and standardised rate ratio in 1,622 municipalities with adequate reporting of vital statistics (2000–2013). (DOCX)

S2 Table Sensitivity analysis: Different classifications of Estratégia de Saúde da Família coverage. (DOCX)

S3 Table Sensitivity analysis: Mortality from accidents. (DOCX)

S4 Table Comparison of ambulatory-care-sensitive conditions and mortality amenable to primary care. (DOCX)

S1 Text Methods for redistribution of ill-defined deaths. (DOCX)

S2 Text Methods for imputing missing race. (DOCX)

S3 Text Fixed-effects longitudinal Poisson regression model specification. (DOCX)

S4 Text Calculating statistical difference in coefficients between two different models. (DOCX)

## Abbreviations

ACSC: ambulatory-care-sensitive condition
COPD: chronic obstructive pulmonary disease
ESF: Estratégia de Saúde da Família
GDP: gross domestic product
PHC: primary healthcare
R$100s: hundreds of Brazilian reais
RR: rate ratio
SDG: Sustainable Development Goal
SRR: standardised rate ratio
UHC: universal health coverage
