# Supplementary material for: Association between expansion of primary healthcare and racial inequalities in mortality amenable to primary care in Brazil: A national longitudinal analysis
Source: PLoS Med. 2017 May 30;14(5):e1002306. doi: 10.1371/journal.pmed.1002306 (PMC5448733; doi:10.1371/journal.pmed.1002306)
Supplement: S1 Alternative Language Abstract — (DOCX) [file pmed.1002306.s001.docx]

**Associação entre expansão da atenção primária à saúde e desigualdades raciais na mortalidade evitável pela atenção primária no Brasil: uma análise longitudinal nacional**

**Resumo**

**Introdução**

A Cobertura Universal de Saúde pode desempenhar um papel importante na consecução do Objetivo de Desenvolvimento Sustentável (ODS) número 10, referente à redução das desigualdades, mas poucos dados de apoio estão disponíveis em países de baixa e média renda. A Estratégia de Saúde da Família (ESF) é um programa brasileiro de atenção primária à saúde, de base comunitária, que vem se expandindo desde a década de 1990 e é a principal plataforma para ofertar cobertura universal à saúde no país. Sendo assim, avaliamos se a expansão da ESF esteve associada a reduções distintas na mortalidade evitável pela atenção primária à saúde (APS) entre grupos raciais.

**Métodos e Resultados**

Utilizou-se regressão longitudinal de painel com efeito fixo, no nível do município, para analisar a associação entre cobertura da ESF e mortalidade por condições sensíveis à atenção primária (CSAP), tanto em pessoas pretas e pardas como brancas, no período de 2000 a 2013. Os modelos foram ajustados por desenvolvimento socioeconômico, municipal e variáveis gerais do sistema de saúde. No período de 2000 a 2013, houve 281.877 mortes por CSAP entre pessoas pretas e pardas e 318.030 entre brancas, nos 1.622 municípios estudados. A mortalidade por CSAP padronizada por idade caiu de 93,3 para 57,9 por 100.000 habitantes entre pessoas pretas e pardas e de 75,7 para 49,2 por 100.000 entre pessoas brancas. A expansão da ESF (de 0 a 100%) foi associada a uma redução de 15,4% (RR: 0,846; IC95%: 0,796 - 0,899) na mortalidade por CSAP entre pessoas pretas e pardas e de 6,8% (RR: 0,932; IC95%: 0,892 - 0,974) entre pessoas brancas (coeficientes significativamente diferentes com valor-p = 0,012). Esses benefícios diferenciais devem-se principalmente por maiores reduções na mortalidade por doenças infecciosas, deficiências nutricionais e anemia, diabetes e doenças cardiovasculares entre as pessoas pretas e pardas. Embora seja uma análise ecológica, as análises de sensibilidade sugerem que mais de 30% dos óbitos entre pessoas pretas e pardas teriam que ser incorretamente codificados para que os resultados fossem inválidos. Uma das limitações deste estudo é o uso de dados municipais agregados, o que impede a inferência no nível individual. Viés de variáveis omitidas, em que fatores associados com a expansão da ESF estão também associados às mudanças nas taxas de mortalidade, pode ter influenciado os resultados, embora as análises de sensibilidade mostrem a robustez dos achados em relação às tendências temporais antes da implantação da ESF e à inclusão de outros atributos municipais que poderiam estar associados à cobertura.

**Conclusão**

A expansão da APS está associada a reduções nas desigualdades de mortalidade entre grupos raciais no Brasil. Esses achados destacam a importância do investimento em APS para alcançar os objetivos de saúde e desigualdade dos ODS.
