# Supplementary material for: Association between expansion of primary healthcare and racial inequalities in mortality amenable to primary care in Brazil: A national longitudinal analysis
Source: PLoS Med. 2017 May 30;14(5):e1002306. doi: 10.1371/journal.pmed.1002306 (PMC5448733; doi:10.1371/journal.pmed.1002306)
Supplement: S1 Appendix — (DOCX) [file pmed.1002306.s002.docx]

**S1 Appendix – Expansion of the Estratégia de Saúde da Família by municipal population, poverty rate, and black/*pardo* population.**

Larger municipalities, those with lower poverty rates, and those with lower proportions of the population classifying as black or *pardo* exhibited slower uptake of the ESF over the period 1998-2013.

**Fig A - The percentage of municipalities reaching 50% ESF coverage by municipal population (in 2000) for the years 2000-2013**

*
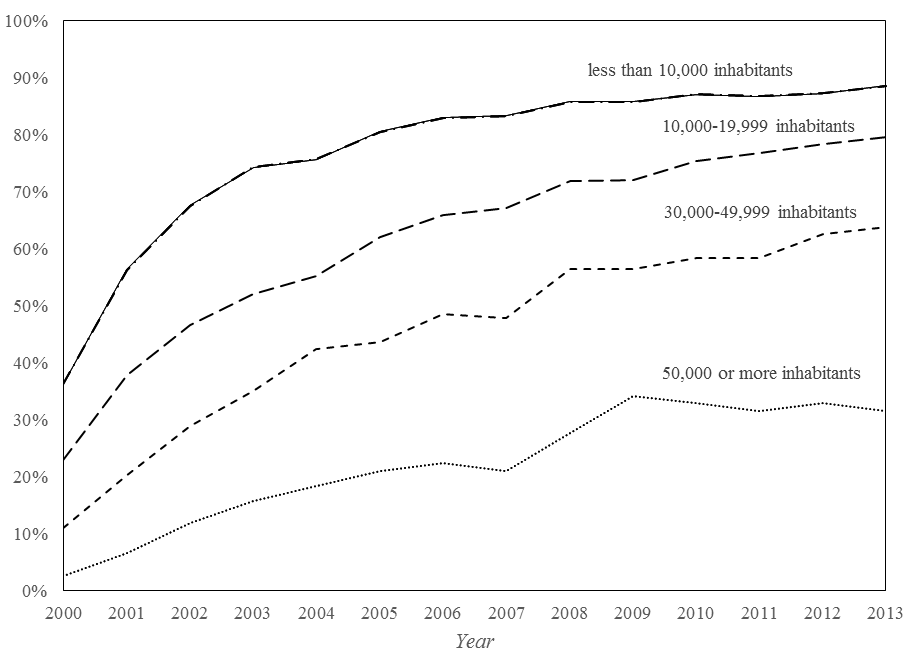
*

**Fig B - The percentage of municipalities reaching 50% ESF coverage by municipal poverty rate (in 2000) for the years 2000-2013**

**
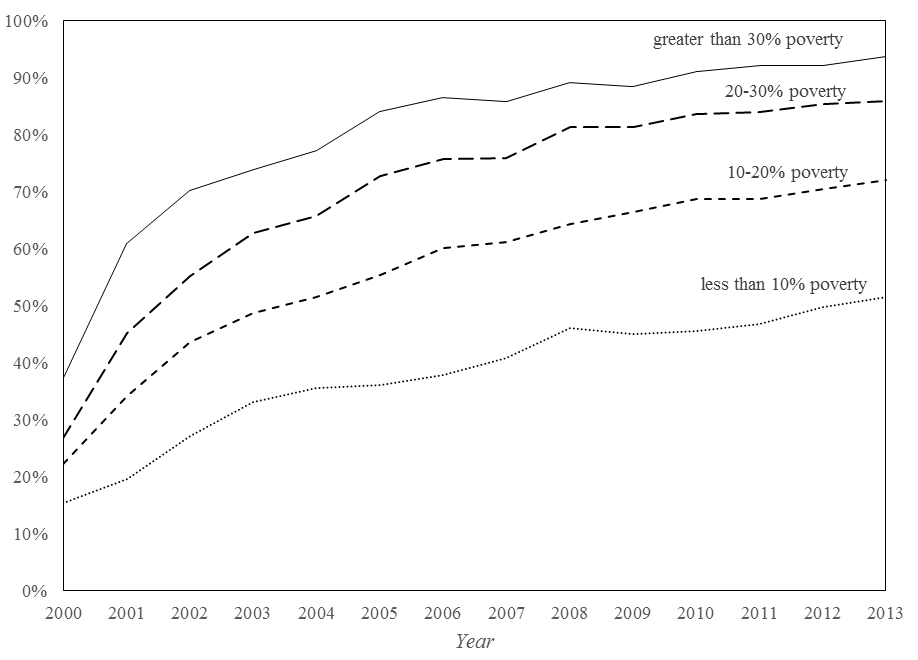
**

Note: Poverty rate is defined as the per capita household income of less than R$255 (in 2010 R$);

**Fig C - The percentage of municipalities reaching 50% ESF coverage by municipal poverty rate (in 2000) for the years 2000-2013**

**
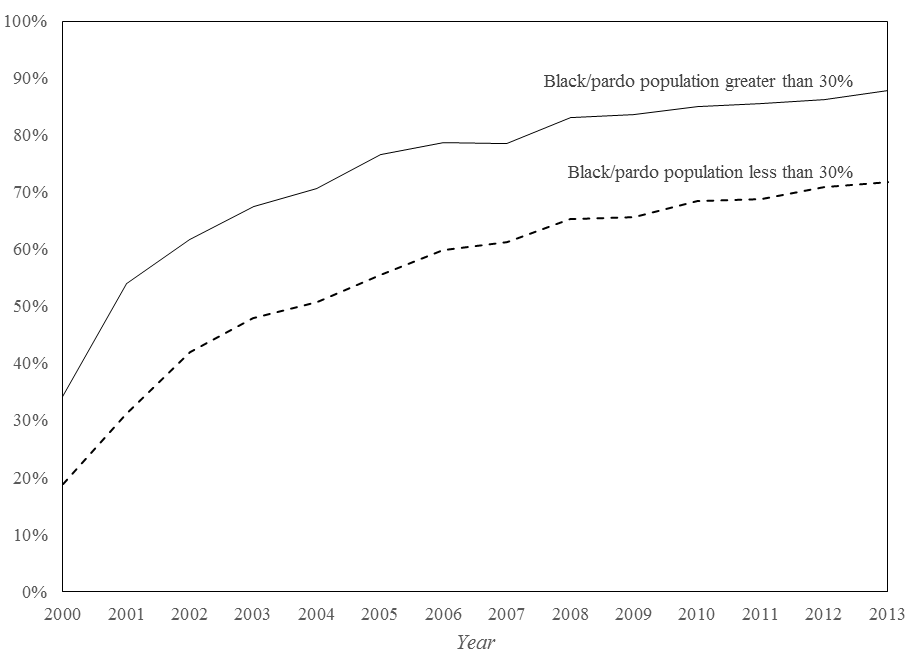
**
