# Supplementary material for: Association between expansion of primary healthcare and racial inequalities in mortality amenable to primary care in Brazil: A national longitudinal analysis
Source: PLoS Med. 2017 May 30;14(5):e1002306. doi: 10.1371/journal.pmed.1002306 (PMC5448733; doi:10.1371/journal.pmed.1002306)
Supplement: S2 Appendix — (DOCX) [file pmed.1002306.s003.docx]

**S2 Appendix - Sensitivity analysis: Sequential addition of covariates and alternative model specifications**

The following two tables demonstrate robustness of the models and findings. The ACSC mortality rates for the black/*pardo* and white populations are examined separately, but the same model specifications are employed for each outcome:

M1 – Random-effects Poisson longitudinal model with ESF coverage and year;

M2 – Fixed-effects Poisson longitudinal model with ESF coverage and year;

M3 – Fixed-effects Poisson longitudinal model with ESF coverage and year + socio-economic factors (Bolsa Família coverage, illiteracy, poverty, and urbanisation);

M4 – Fixed-effects Poisson longitudinal model with ESF coverage and year + socio-economic factors + wider health system factors (public healthcare spending, public hospital beds, and private hospital beds);

M5 – Fixed-effects Poisson longitudinal model with ESF coverage and year + socio-economic factors + wider health system factors + private insurance and wealth (private healthcare insurance, GDP and a (Private healthcare insurance) x (GDP) interaction) (The same fully-adjusted model as presented in the main paper);

M6 – Fixed-effects Poisson longitudinal model (fully-adjusted (same as M5)) + State-year fixed effects (not reported);

M7 – Fixed-effects Negative Binomial longitudinal model (fully-adjusted (same as M5));

The statistical significance between the ESF coverage coefficients for the respective black/*pardo* and white populations models were calculated using the methods specified in S4 Table. For the random-effects model (M1), the coefficients were not significantly different. For models M2 to M7, the coefficients were significantly different at p=0.045, p=0.023, p=0.016, p=0.012, p=0.010, and p=0.012 respectively.

**Table A - Results from longitudinal regressions of ACSC mortality in the black/*pardo* population with varying model specifications**

|  | **M1 (RE)** | | **M2 (FE)** | | | | **M3 (FE)** | | | | **M4 (FE)** | | | | **M5 (FE)** | | | | | **M6 (FE + State)** | | | | | **M7 (NB)** | | | | | |
| --- | --- | --- | --- | --- | --- | --- | --- | --- | --- | --- | --- | --- | --- | --- | --- | --- | --- | --- | --- | --- | --- | --- | --- | --- | --- | --- | --- | --- | --- | --- |
|  | **RR** | **95% CI** | **RR** | **95% CI** | | **RR** | | | **95% CI** | **RR** | | **95% CI** | | **RR** | | **95% CI** | | **RR** | | | **95% CI** | | **RR** | | | **95% CI** | | |  |  |
| ESF coverage | 0.906** | 0.850,0.965 | 0.873*** | 0.825,0.923 | | 0.867*** | | | 0.818,0.918 | 0.861*** | | 0.812,0.912 | | 0.846*** | | 0.796,0.899 | | 0.842*** | | | 0.793,0.895 | | 0.856*** | | | 0.824,0.889 | | |  |  |
| Year | 0.968*** | 0.965,0.971 | 0.969*** | 0.966,0.972 | | 0.974*** | | | 0.962,0.986 | 0.972*** | | 0.960,0.985 | | 0.966*** | | 0.954,0.979 | | 0.968 | | | 0.931,1.006 | | 0.995 | | | 0.989,1.001 | | |  |  |
| Bolsa Família coverage | - | - | - | - | | 0.952 | | | 0.853,1.063 | 0.958 | | 0.860,1.067 | | 0.873* | | 0.784,0.973 | | 0.993 | | | 0.892,1.104 | | 0.823*** | | | 0.756,0.896 | | |  |  |
| Illiteracy | - | - | - | - | | 1.106 | | | 0.915,1.337 | 1.123 | | 0.923,1.366 | | 0.94 | | 0.757,1.168 | | 0.994 | | | 0.785,1.258 | | 1.552*** | | | 1.408,1.710 | | |  |  |
| Poverty | - | - | - | - | | 0.99 | | | 0.655,1.496 | 0.978 | | 0.637,1.503 | | 1.592* | | 1.053,2.407 | | 1.035 | | | 0.676,1.585 | | 1.462*** | | | 1.186,1.802 | | |  |  |
| Urbanisation | - | - | - | - | | 1.632 | | | 0.913,2.918 | 1.607 | | 0.909,2.841 | | 1.135 | | 0.697,1.848 | | 0.89 | | | 0.547,1.448 | | 0.696** | | | 0.542,0.893 | | |  |  |
| Public healthcare spending | - | - | - | - | | - | | | - | 1.007 | | 0.998,1.017 | | 1.009 | | 1.000,1.019 | | 1.007 | | | 0.999,1.014 | | 1.014*** | | | 1.008,1.020 | | |  |  |
| Public hospital beds | - | - | - | - | | - | | | - | 0.992 | | 0.919,1.071 | | 1.001 | | 0.941,1.065 | | 1.013 | | | 0.952,1.077 | | 1.032 | | | 0.978,1.089 | | |  |  |
| Private hospital beds | - | - | - | - | | - | | | - | 1.096 | | 0.837,1.435 | | 1.193 | | 0.913,1.561 | | 1.114 | | | 0.891,1.393 | | 1.083 | | | 0.929,1.262 | | |  |  |
| Private healthcare insurance | - | - | - | - | | - | | | - | - | | - | | 0.831** | | 0.744,0.928 | | 0.907* | | | 0.822,1.000 | | 0.851*** | | | 0.801,0.903 | | |  |  |
| GDP | - | - | - | - | | - | | | - | - | | - | | 0.846** | | 0.759,0.944 | | 0.933 | | | 0.847,1.029 | | 0.845*** | | | 0.792,0.902 | | |  |  |
| (Private healthcare insurance) x (GDP) | - | - | - | - | | - | | | - | - | | - | | 0.953*** | | 0.934,0.972 | | 0.972** | | | 0.955,0.990 | | 0.962*** | | | 0.951,0.972 | | |  |  |
|  |  |  |  |  | |  | | |  |  | |  | |  | |  | |  | | |  | |  | | |  | | |  |  |
| N (Observations) | 22,705 |  | 22,384 |  | | 22,384 | | |  | 22,384 | |  | | 22,384 | |  | | 22,384 | | |  | | 22,384 | | |  | | |  |  |
| N (Municipalities) | 1,622 |  | 1,599 |  | | 1,599 | | |  | 1,599 | |  | | 1,599 | |  | | 1,599 | | |  | | 1,599 | | |  | | |  |  |
|  |  |  |  | |  | | |  |  | |  | |  | |  | |  | |  | | |  | |  | | |  | | |  |
| Exponentiated coefficients;* p<0.05, ** p<0.01, *** p<0.001 RR- Rate Ratio; 95% CI- 95% confidence interval; ESF - Estratégia de Saúde da Família (Family Health Strategy); GDP – Gross Domestic Product;  Notes: The study period was from 2000 to 2013. Robust standards errors employed in all models except M7 (negative binomial). Year is a continuous variable and is interpreted as the change in mortality rate for each additional year. ESF coverage is a two year average of within year municipal ESF coverage and coverage in the year before. ESF coverage, Bolsa Família coverage, poverty rate and the urbanisation rate are all expressed as percentages and scaled so a 1 unit increase represents a 100% increase. Private healthcare insurance is also expressed as a percentage, but is log transformed. Illiteracy is the illiteracy rate of those aged 25 and over and is log transformed. Public healthcare spending is expressed as R$100s per person as is GDP, although GDP is log transformed. Public and private hospital beds are expressed per 1,000 municipal inhabitants. Some municipalities and/or year observations not included due to no deaths from ambulatory care sensitive conditions for that racial group. M1 is random-effects Poisson model. M2-M6 are fixed-effects Poisson models. Model M6 additionally controls for State-year fixed-effects. M7 is a fixed-effect negative binomial regression. | | | | | | | | | | | | | | | | | | | | | | | | | | | |  |  |  |

**Table B - Results from longitudinal regressions of ACSC mortality in the white population with varying model specifications**

|  | **M1 (RE)** | | **M2 (FE)** | | | | **M3 (FE)** | | | | **M4 (FE)** | | | | **M5 (FE)** | | | | | **M6 (FE + State)** | | | | | **M7 (NB)** | | | | | |
| --- | --- | --- | --- | --- | --- | --- | --- | --- | --- | --- | --- | --- | --- | --- | --- | --- | --- | --- | --- | --- | --- | --- | --- | --- | --- | --- | --- | --- | --- | --- |
|  | **RR** | **95% CI** | **RR** | **95% CI** | | **RR** | | | **95% CI** | **RR** | | **95% CI** | | **RR** | | **95% CI** | | **RR** | | | **95% CI** | | **RR** | | | **95% CI** | | |  |  |
| ESF coverage | 0.929*** | 0.897,0.963 | 0.937** | 0.899,0.978 | | 0.943** | | | 0.903,0.984 | 0.942** | | 0.901,0.985 | | 0.932** | | 0.892,0.974 | | 0.929*** | | | 0.889,0.970 | | 0.933*** | | | 0.906,0.960 | | |  |  |
| Year | 0.969*** | 0.967,0.971 | 0.969*** | 0.966,0.971 | | 0.966*** | | | 0.960,0.973 | 0.966*** | | 0.959,0.973 | | 0.971*** | | 0.963,0.979 | | 0.949*** | | | 0.929,0.969 | | 0.970*** | | | 0.965,0.976 | | |  |  |
| Bolsa Família coverage | - | - | - | - | | 0.932 | | | 0.838,1.035 | 0.928 | | 0.833,1.034 | | 0.895 | | 0.799,1.002 | | 0.912 | | | 0.817,1.019 | | 0.878** | | | 0.808,0.954 | | |  |  |
| Illiteracy | - | - | - | - | | 0.954 | | | 0.843,1.079 | 0.947 | | 0.833,1.077 | | 0.905 | | 0.796,1.029 | | 0.901 | | | 0.798,1.018 | | 0.888** | | | 0.815,0.968 | | |  |  |
| Poverty | - | - | - | - | | 0.89 | | | 0.687,1.151 | 0.879 | | 0.679,1.138 | | 1.149 | | 0.849,1.555 | | 0.952 | | | 0.687,1.320 | | 1.056 | | | 0.865,1.288 | | |  |  |
| Urbanisation | - | - | - | - | | 0.925 | | | 0.636,1.345 | 0.925 | | 0.634,1.351 | | 0.839 | | 0.585,1.204 | | 0.814 | | | 0.590,1.124 | | 0.764* | | | 0.604,0.965 | | |  |  |
| Public healthcare spending | - | - | - | - | | - | | | - | 0.999 | | 0.987,1.011 | | 1.002 | | 0.991,1.013 | | 1.002 | | | 0.991,1.013 | | 1.003 | | | 0.998,1.008 | | |  |  |
| Public hospital beds | - | - | - | - | | - | | | - | 1.009 | | 0.954,1.069 | | 1.013 | | 0.960,1.069 | | 1.011 | | | 0.961,1.063 | | 1.015 | | | 0.975,1.057 | | |  |  |
| Private hospital beds | - | - | - | - | | - | | | - | 1.056 | | 0.897,1.243 | | 1.088 | | 0.922,1.284 | | 1.068 | | | 0.928,1.230 | | 1.095 | | | 0.983,1.219 | | |  |  |
| Private healthcare insurance | - | - | - | - | | - | | | - | - | | - | | 0.900* | | 0.827,0.979 | | 0.891** | | | 0.828,0.960 | | 0.892*** | | | 0.846,0.941 | | |  |  |
| GDP | - | - | - | - | | - | | | - | - | | - | | 0.853*** | | 0.781,0.932 | | 0.867*** | | | 0.801,0.939 | | 0.845*** | | | 0.800,0.893 | | |  |  |
| (Private healthcare insurance) x (GDP) | - | - | - | - | | - | | | - | - | | - | | 0.974** | | 0.959,0.990 | | 0.974*** | | | 0.960,0.989 | | 0.973*** | | | 0.963,0.983 | | |  |  |
|  |  |  |  |  | |  | | |  |  | |  | |  | |  | |  | | |  | |  | | |  | | |  |  |
| N (Observations) | 22,708 |  | 22,694 |  | | 22,694 | | |  | 22,694 | |  | | 22,694 | |  | | 22,694 | | |  | | 22,694 | | |  | | |  |  |
| N (Municipalities) | 1,622 |  | 1,621 |  | | 1,621 | | |  | 1,621 | |  | | 1,621 | |  | | 1,621 | | |  | | 1,621 | | |  | | |  |  |
|  |  |  |  | |  | | |  |  | |  | |  | |  | |  | |  | | |  | |  | | |  | | |  |
| Exponentiated coefficients;* p<0.05, ** p<0.01, *** p<0.001 RR- Rate Ratio; 95% CI- 95% confidence interval; ESF - Estratégia de Saúde da Família (Family Health Strategy); GDP – Gross Domestic Product;  Notes: The study period was from 2000 to 2013. Robust standards errors employed in all models except M7 (negative binomial). ESF coverage is a two year average of within year municipal ESF coverage and coverage in the year before. Year is a continuous variable and is interpreted as the underlying annual change in mortality rate during the study period. ESF coverage, Bolsa Família coverage, poverty rate and the urbanisation rate are all expressed as percentages and scaled so a 1 unit increase represents a 100% increase. Private healthcare insurance is also expressed as a percentage, but is log transformed. Illiteracy is the illiteracy rate of those aged 25 and over and is log transformed. Public healthcare spending is expressed as R$100s per person as is GDP, although GDP is log transformed. Public and private hospital beds are expressed per 1,000 municipal inhabitants. Some municipalities and/or year observations not included due to no deaths from ambulatory care sensitive conditions for that racial group. M1 is random-effects Poisson model. M2-M6 are fixed-effects Poisson models. Model M6 additionally controls for State-year fixed-effects. M7 is a fixed-effect negative binomial regression. | | | | | | | | | | | | | | | | | | | | | | | | | | | |  |  |  |
