# Supplementary material for: Association between expansion of primary healthcare and racial inequalities in mortality amenable to primary care in Brazil: A national longitudinal analysis
Source: PLoS Med. 2017 May 30;14(5):e1002306. doi: 10.1371/journal.pmed.1002306 (PMC5448733; doi:10.1371/journal.pmed.1002306)
Supplement: S5 Appendix — (DOCX) [file pmed.1002306.s006.docx]

**S5 Appendix – Sensitivity analysis: analysing all municipalities (including those with inadequate vital statistics reporting)**

The analyses were repeated using all municipalities and not just those defined as having adequate vital statistics reporting.

**Table A - Results from longitudinal fixed-effects Poisson regression of ACSC mortality in black/ *pardo* and white populations in all municipalities (including those with inadequate vital statistics reporting)**

|  | **Black and *Pardo*** | | **White** | |
| --- | --- | --- | --- | --- |
|  | **RR** | **95% CI** | **RR** | **95% CI** |
| ESF coverage | 0.973 | 0.940,1.008 | 0.925*** | 0.896,0.955 |
| Year | 0.963*** | 0.954,0.971 | 0.965*** | 0.959,0.972 |
| Bolsa Família coverage | 1.012 | 0.957,1.069 | 1.007 | 0.944,1.075 |
| Illiteracy | 1.011 | 0.882,1.159 | 0.897* | 0.811,0.992 |
| Poverty | 1.429** | 1.135,1.799 | 1.070 | 0.866,1.321 |
| Urbanisation | 1.554** | 1.166,2.072 | 0.981 | 0.776,1.240 |
| Public healthcare spending | 1.019*** | 1.011,1.027 | 1.005 | 0.996,1.013 |
| Public hospital beds | 0.984 | 0.930,1.041 | 1.062* | 1.009,1.117 |
| Private hospital beds | 0.929 | 0.759,1.138 | 0.968 | 0.855,1.095 |
| Private healthcare insurance | 0.809*** | 0.756,0.865 | 0.888*** | 0.840,0.938 |
| GDP | 0.824*** | 0.770,0.882 | 0.874*** | 0.823,0.929 |
| (Private healthcare insurance) x (GDP) | 0.952*** | 0.942,0.962 | 0.969*** | 0.959,0.979 |
|  |  |  |  |  |
| N (Observations) | 75,047 |  | 76,583 |  |
| N (Municipalities) | 5,361 |  | 5,471 |  |

Exponentiated coefficients; * p<0.05, ** p<0.01, *** p<0.001 RR- Rate Ratio; 95% CI- 95% confidence interval; ESF - Estratégia de Saúde da Família (Family Health Strategy); GDP – Gross Domestic Product;

Notes: The study period was from 2000 to 2013. Robust standards errors employed. ESF coverage is a two year average of within year municipal ESF coverage and coverage in the year before. Year is a continuous variable and is interpreted as the underlying annual change in mortality rate during the study period. ESF coverage, Bolsa Família coverage, poverty rate and the urbanisation rate are all expressed as percentages and scaled so a 1 unit increase represents a 100% increase. Private healthcare insurance is also expressed as a percentage, but is log transformed. Illiteracy is the illiteracy rate of those aged 25 and over and is log transformed. Public healthcare spending is expressed as R$100s per person as is GDP, although GDP is log transformed. Public and private hospital beds are expressed per 1,000 municipal inhabitants. Some municipalities and/or year observations not included due to no deaths from ambulatory care sensitive conditions for that racial group.

**Table B - Results from longitudinal fixed-effects linear regression, with Standardised Rate Ratio for mortality from ACSCs in black/ *pardo* and white populations in all municipalities (including those with inadequate vital statistics reporting)**

|  | **Coeff.** | **95% CI** | |
| --- | --- | --- | --- |
| ESF coverage | -0.166** | -0.285,-0.047 | |
| Year | 0.008 | -0.020, 0.037 | |
| Bolsa Família coverage | -0.018 | -0.253, 0.217 | |
| Illiteracy | 0.012 | -0.476, 0.501 | |
| Poverty | 0.662 | -0.126, 1.451 | |
| Urbanisation | 0.269 | -0.575, 1.113 | |
| Public healthcare spending | 0.014 | -0.025, 0.052 | |
| Public hospital beds | -0.034 | -0.174, 0.105 | |
| Private hospital beds | -0.035 | -0.433, 0.363 | |
| Private healthcare insurance | 0.109 | -0.100, 0.319 | |
| GDP | -0.084 | -0.307, 0.138 | |
| (Private healthcare insurance) x (GDP) | 0.009 | -0.027, 0.044 | |
|  |  |  | |
| N (Observations) | 21,238 |  | |
| N (Municipalities) | 5,507 |  | |
| * p<0.05, ** p<0.01, *** p<0.001 Coeff – Coefficient; 95% CI- 95% confidence interval; ESF - Estratégia de Saúde da Família (Family Health Strategy); GDP – Gross Domestic Product;  Notes: The study period was from 2000 to 2013. Robust standards errors employed. Year is a continuous variable and is interpreted as the change in mortality rate for each additional year. ESF coverage is a two year average of within year municipal ESF coverage and coverage in the year before. ESF coverage, Bolsa Família coverage, poverty rate and the urbanisation rate are all expressed as percentages and scaled so a 1 unit increase represents a 100% increase. Private healthcare insurance is also expressed as a percentage, but is log transformed. Illiteracy is the illiteracy rate of those aged 25 and over and is log transformed. Public healthcare spending is expressed as R$100s per person as is GDP, although GDP is log transformed. Public and private hospital beds are expressed per 1,000 municipal inhabitants. Some municipalities and/or year observations not included due to no deaths from ambulatory care sensitive conditions for that racial group. | | |  |
|  | | |  |
