# Supplementary material for: Association between expansion of primary healthcare and racial inequalities in mortality amenable to primary care in Brazil: A national longitudinal analysis
Source: PLoS Med. 2017 May 30;14(5):e1002306. doi: 10.1371/journal.pmed.1002306 (PMC5448733; doi:10.1371/journal.pmed.1002306)
Supplement: S6 Appendix — (DOCX) [file pmed.1002306.s007.docx]

**S6 Appendix – Sensitivity analysis: testing potential misclassification** **bias**

Potential misclassification bias of race on death certificates could occur as, whilst population data is self-reported, death certificate data is not. The effect of misclassification of race on the results was tested by reclassifying 0%, 10%, 20%, and 30% of black/*pardo* deaths as white deaths and repeating the analysis.

Identical multiple regression models as the main analysis are employed, but covariates omitted for clarity.

**Table A - Results from longitudinal fixed-effects Poisson regression of ACSC mortality in black/ *pardo* and white populations with no reclassification of black/*pardo* deaths**

|  | **Black and *Pardo*** | | **White** | |
| --- | --- | --- | --- | --- |
|  | **RR** | **95% CI** | **RR** | **95% CI** |
| ESF coverage | 0.873** | 0.804,0.948 | 0.932*** | 0.898,0.969 |
| Year | 0.967*** | 0.950,0.985 | 0.975*** | 0.966,0.985 |
| N (Observations) | 22,384 |  | 22,694 |  |
| N (Municipalities) | 1,599 |  | 1,621 |  |

*ESF coefficients significantly different (p=0.011) (results reported in main analysis)*

Exponentiated coefficients; * p<0.05, ** p<0.01, *** p<0.001 RR- Rate Ratio; 95% CI- 95% confidence interval; ESF - Estratégia de Saúde da Família (Family Health Strategy);

Notes: The study period was from 2000 to 2013. Robust standards errors employed. ESF coverage is a two year average of within year municipal ESF coverage and coverage in the year before, and is expressed as percentages and scaled so a 1 unit increase represents a 100% increase. Year is a continuous variable and is interpreted as the underlying annual change in mortality rate during the study period. Although not reported, all regressions control for: Bolsa *Família* coverage (%); Illiteracy rate of those over 25 (Log); Poverty rate (%); Urban rate (%); Public Healthcare spending (R$100 per person); Public hospital beds per 1,000 population; Private hospital beds per 1,000 population; Private healthcare insurance (%) (Log); GDP per person (R$100) (Log); Private healthcare insurance (%) (Log)* GDP per person (R$100) (Log) Interaction. Some municipalities and/or year observations not included due to no deaths from ambulatory care sensitive conditions for that racial group.

**Table B - Results from longitudinal fixed-effects Poisson regression of ACSC mortality in black/ *pardo* and white populations with 10% of black/*pardo* deaths reclassified as white deaths**

|  | **Black and *Pardo*** | | **White** | |
| --- | --- | --- | --- | --- |
|  | **RR** | **95% CI** | **RR** | **95% CI** |
| ESF coverage | 0.846*** | 0.797,0.899 | 0.921*** | 0.884,0.959 |
| Year | 0.967*** | 0.954,0.979 | 0.972*** | 0.964,0.980 |
| N (Observations) | 22,384 |  | 22,694 |  |
| N (Municipalities) | 1,599 |  | 1,621 |  |

*ESF coefficients significantly different (p=0.023)*

Exponentiated coefficients; * p<0.05, ** p<0.01, *** p<0.001 RR- Rate Ratio; 95% CI- 95% confidence interval; ESF - Estratégia de Saúde da Família (Family Health Strategy);

Notes: The study period was from 2000 to 2013. Robust standards errors employed. ESF coverage is a two year average of within year municipal ESF coverage and coverage in the year before, and is expressed as percentages and scaled so a 1 unit increase represents a 100% increase. Year is a continuous variable and is interpreted as the underlying annual change in mortality rate during the study period. Although not reported, all regressions control for: Bolsa *Família* coverage (%); Illiteracy rate of those over 25 (Log); Poverty rate (%); Urban rate (%); Public Healthcare spending (R$100 per person); Public hospital beds per 1,000 population; Private hospital beds per 1,000 population; Private healthcare insurance (%) (Log); GDP per person (R$100) (Log); Private healthcare insurance (%) (Log)* GDP per person (R$100) (Log) Interaction. Some municipalities and/or year observations not included due to no deaths from ambulatory care sensitive conditions for that racial group.

**Table C - Results from longitudinal fixed-effects Poisson regression of ACSC mortality in black/ *pardo* and white populations with 20% of black/*pardo* deaths reclassified as white deaths**

|  | **Black and *Pardo*** | | **White** | |
| --- | --- | --- | --- | --- |
|  | **RR** | **95% CI** | **RR** | **95% CI** |
| ESF coverage | 0.847*** | 0.797,0.900 | 0.912*** | 0.878,0.948 |
| Year | 0.966*** | 0.954,0.978 | 0.973*** | 0.965,0.981 |
| N (Observations) | 22,384 |  | 22,694 |  |
| N (Municipalities) | 1,599 |  | 1,621 |  |

*ESF coefficients significantly different (p=0.043)*

Exponentiated coefficients; * p<0.05, ** p<0.01, *** p<0.001 RR- Rate Ratio; 95% CI- 95% confidence interval; ESF - Estratégia de Saúde da Família (Family Health Strategy);

Notes: The study period was from 2000 to 2013. Robust standards errors employed. ESF coverage is a two year average of within year municipal ESF coverage and coverage in the year before, and is expressed as percentages and scaled so a 1 unit increase represents a 100% increase. Year is a continuous variable and is interpreted as the underlying annual change in mortality rate during the study period. Although not reported, all regressions control for: Bolsa *Família* coverage (%); Illiteracy rate of those over 25 (Log); Poverty rate (%); Urban rate (%); Public Healthcare spending (R$100 per person); Public hospital beds per 1,000 population; Private hospital beds per 1,000 population; Private healthcare insurance (%) (Log); GDP per person (R$100) (Log); Private healthcare insurance (%) (Log)* GDP per person (R$100) (Log) Interaction. Some municipalities and/or year observations not included due to no deaths from ambulatory care sensitive conditions for that racial group.

**Table D - Results from longitudinal fixed-effects Poisson regression of ACSC mortality in black/ *pardo* and white populations with 30% of black/*pardo* deaths reclassified as white deaths**

|  | **Black and *Pardo*** | | **White** | |
| --- | --- | --- | --- | --- |
|  | **RR** | **95% CI** | **RR** | **95% CI** |
| ESF coverage | 0.844*** | 0.794,0.897 | 0.906*** | 0.873,0.941 |
| Year | 0.967*** | 0.955,0.979 | 0.974*** | 0.966,0.983 |
| N (Observations) | 22,384 |  | 22,694 |  |
| N (Municipalities) | 1,599 |  | 1,621 |  |

*ESF coefficients significantly different (p=0.05)*

| Exponentiated coefficients; * p<0.05, ** p<0.01, *** p<0.001 RR- Rate Ratio; 95% CI- 95% confidence interval; ESF - Estratégia de Saúde da Família (Family Health Strategy);  Notes: The study period was from 2000 to 2013. Robust standards errors employed. ESF coverage is a two year average of within year municipal ESF coverage and coverage in the year before, and is expressed as percentages and scaled so a 1 unit increase represents a 100% increase. Year is a continuous variable and is interpreted as the underlying annual change in mortality rate during the study period. Although not reported, all regressions control for: Bolsa *Família* coverage (%); Illiteracy rate of those over 25 (Log); Poverty rate (%); Urban rate (%); Public Healthcare spending (R$100 per person); Public hospital beds per 1,000 population; Private hospital beds per 1,000 population; Private healthcare insurance (%) (Log); GDP per person (R$100) (Log); Private healthcare insurance (%) (Log)* GDP per person (R$100) (Log) Interaction. Some municipalities and/or year observations not included due to no deaths from ambulatory care sensitive conditions for that racial group. | | | | |
| --- | --- | --- | --- | --- |
|  |  |  |  |  |

**Table E - Results from longitudinal fixed-effects linear regression of the ACSC mortality SRR between the black/ *pardo* and white populations no reclassification of black/*pardo* deaths**

|  | **Coeff.** | **95% CI** |
| --- | --- | --- |
| ESF coverage | -0.179* | -0.336,-0.022 |
| Year | 0.010 | -0.021, 0.041 |
| N (Observations) | 21,238 |  |
| N (Municipalities) | 5,507 |  |

* p<0.05, ** p<0.01, *** p<0.001 Coeff – Coefficient; 95% CI- 95% confidence interval; ESF - Estratégia de Saúde da Família (Family Health Strategy);

Notes: The study period was from 2000 to 2013. Robust standards errors employed. ESF coverage is a two year average of within year municipal ESF coverage and coverage in the year before, and is expressed as percentages and scaled so a 1 unit increase represents a 100% increase. Year is a continuous variable and is interpreted as the underlying annual change in mortality rate during the study period. Although not reported, all regressions control for: Bolsa *Família* coverage (%); Illiteracy rate of those over 25 (Log); Poverty rate (%); Urban rate (%); Public Healthcare spending (R$100 per person); Public hospital beds per 1,000 population; Private hospital beds per 1,000 population; Private healthcare insurance (%) (Log); GDP per person (R$100) (Log); Private healthcare insurance (%) (Log)* GDP per person (R$100) (Log) Interaction. Some municipalities and/or year observations not included due to no deaths from ambulatory care sensitive conditions for that racial group.

**Table F - Results from longitudinal fixed-effects linear regression of the ACSC mortality SRR between the black/ *pardo* and white populations with 10% of black/*pardo* deaths reclassified as white**

|  | **Coeff.** | **95% CI** |
| --- | --- | --- |
| ESF coverage | -0.086* | -0.157,-0.015 |
| Year | 0.005 | -0.010, 0.020 |
| N (Observations) | 21,238 |  |
| N (Municipalities) | 5,507 |  |

* p<0.05, ** p<0.01, *** p<0.001 Coeff – Coefficient; 95% CI- 95% confidence interval; ESF - Estratégia de Saúde da Família (Family Health Strategy);

Notes: The study period was from 2000 to 2013. Robust standards errors employed. ESF coverage is a two year average of within year municipal ESF coverage and coverage in the year before, and is expressed as percentages and scaled so a 1 unit increase represents a 100% increase. Year is a continuous variable and is interpreted as the underlying annual change in mortality rate during the study period. Although not reported, all regressions control for: Bolsa *Família* coverage (%); Illiteracy rate of those over 25 (Log); Poverty rate (%); Urban rate (%); Public Healthcare spending (R$100 per person); Public hospital beds per 1,000 population; Private hospital beds per 1,000 population; Private healthcare insurance (%) (Log); GDP per person (R$100) (Log); Private healthcare insurance (%) (Log)* GDP per person (R$100) (Log) Interaction. Some municipalities and/or year observations not included due to no deaths from ambulatory care sensitive conditions for that racial group.

**Table G - Results from longitudinal fixed-effects linear regression of the ACSC mortality SRR between the black/ *pardo* and white populations with 20% of black/*pardo* deaths reclassified as white**

|  | **Coeff.** | **95% CI** |
| --- | --- | --- |
| ESF coverage | -0.054* | -0.100,-0.009 |
| Year | 0.003 | -0.006, 0.013 |
| N (Observations) | 21,238 |  |
| N (Municipalities) | 5,507 |  |

* p<0.05, ** p<0.01, *** p<0.001 Coeff – Coefficient; 95% CI- 95% confidence interval; ESF - Estratégia de Saúde da Família (Family Health Strategy);

Notes: The study period was from 2000 to 2013. Robust standards errors employed. ESF coverage is a two year average of within year municipal ESF coverage and coverage in the year before, and is expressed as percentages and scaled so a 1 unit increase represents a 100% increase. Year is a continuous variable and is interpreted as the underlying annual change in mortality rate during the study period. Although not reported, all regressions control for: Bolsa *Família* coverage (%); Illiteracy rate of those over 25 (Log); Poverty rate (%); Urban rate (%); Public Healthcare spending (R$100 per person); Public hospital beds per 1,000 population; Private hospital beds per 1,000 population; Private healthcare insurance (%) (Log); GDP per person (R$100) (Log); Private healthcare insurance (%) (Log)* GDP per person (R$100) (Log) Interaction. Some municipalities and/or year observations not included due to no deaths from ambulatory care sensitive conditions for that racial group.

**Table H - Results from longitudinal fixed-effects linear regression of the ACSC mortality SRR between the black/ *pardo* and white populations with 30% of black/*pardo* deaths reclassified as white**

|  | **Coeff.** | **95% CI** |
| --- | --- | --- |
| ESF coverage | -0.037* | -0.069,-0.005 |
| Year | 0.003 | -0.004, 0.009 |
| N (Observations) | 21,238 |  |
| N (Municipalities) | 5,507 |  |

* p<0.05, ** p<0.01, *** p<0.001 Coeff – Coefficient; 95% CI- 95% confidence interval; ESF - Estratégia de Saúde da Família (Family Health Strategy);

Notes: The study period was from 2000 to 2013. Robust standards errors employed. ESF coverage is a two year average of within year municipal ESF coverage and coverage in the year before, and is expressed as percentages and scaled so a 1 unit increase represents a 100% increase. Year is a continuous variable and is interpreted as the underlying annual change in mortality rate during the study period. Although not reported, all regressions control for: Bolsa *Família* coverage (%); Illiteracy rate of those over 25 (Log); Poverty rate (%); Urban rate (%); Public Healthcare spending (R$100 per person); Public hospital beds per 1,000 population; Private hospital beds per 1,000 population; Private healthcare insurance (%) (Log); GDP per person (R$100) (Log); Private healthcare insurance (%) (Log)* GDP per person (R$100) (Log) Interaction. Some municipalities and/or year observations not included due to no deaths from ambulatory care sensitive conditions for that racial group.
