# Supplementary material for: Association between expansion of primary healthcare and racial inequalities in mortality amenable to primary care in Brazil: A national longitudinal analysis
Source: PLoS Med. 2017 May 30;14(5):e1002306. doi: 10.1371/journal.pmed.1002306 (PMC5448733; doi:10.1371/journal.pmed.1002306)
Supplement: S7 Appendix — (DOCX) [file pmed.1002306.s008.docx]

**S7 Appendix – Sensitivity analysis: interaction between Bolsa Família and Estratégia de Saúde da Família coverage**

There is evidence synergistic reductions in mortality with ESF and Bolsa Família coverage may exist [1]. This was examined with an interaction between Bolsa Família coverage and ESF coverage. A categorical Bolsa Família variable was employed to aid interpretability of the interaction. These analyses are not meant to robustly evaluate interactions between the Bolsa Família and the ESF given ACSCs may be inappropriate as health outcomes for the Bolsa Família and alternative modelling methods of inequalities may be preferred. Instead they aim to demonstrate the robustness of the findings presented in the main paper.

M1 shows the model which is presented in the main paper including Bolsa Família coverage as a continuous variable. M2 and M3 employ Bolsa Família coverage as categorical variables with M3 also showing an interaction with ESF coverage.

The ESF *Bolsa Família interaction (M3) is non-significant overall at p=0.063 for the black/*pardo* population and is non-significant overall at p=0.760 for the white population. The differences in the ESF coverage coefficients between the black/*pardo* population and white population are significant for M2 and M3 at p=0.015 and p=0.002 respectively.

**Table A - Results from longitudinal fixed-effects Poisson regression of ACSC mortality for the black/ *pardo* population with an interaction between ESF coverage and Bolsa Família coverage**

|  | **M1** | | **M2** | | **M3** | | |  |
| --- | --- | --- | --- | --- | --- | --- | --- | --- |
|  | **RR** | **95% CI** | **RR** | **95% CI** | | **RR** | **95% CI** | |
| ESF coverage | 0.846*** | 0.796,0.899 | 0.856*** | 0.806,0.909 | | 0.817*** | 0.762,0.876 | |
| Year | 0.966*** | 0.954,0.979 | 0.969*** | 0.958,0.981 | | 0.970*** | 0.959,0.982 | |
| Bolsa Família coverage (cont) | 0.873* | 0.784,0.973 | - | - | | - | - | |
| Bolsa Família coverage (cat) |  |  |  |  | |  |  | |
| None (0%) | - | - | 1.000 | Ref | | 1.000 | Ref | |
| Medium (0-20%) | - | - | 0.956** | 0.930,0.983 | | 0.936** | 0.893,0.982 | |
| High (20%+) | - | - | 0.954** | 0.925,0.985 | | 0.930* | 0.872,0.992 | |
| ESF * Bolsa Família coverage |  |  |  |  | |  |  | |
| ESF coverage * None (0%) | - | - | - | - | | 1.000 | Ref | |
| ESF coverage * Med (0-20%) | - | - | - | - | | 1.075 | 0.992,1.166 | |
| ESF coverage * High (20%+) | - | - | - | - | | 1.077 | 0.990,1.170 | |
| Illiteracy | 0.940 | 0.757,1.168 | 0.974 | 0.792,1.198 | | 0.978 | 0.798,1.198 | |
| Poverty | 1.592* | 1.053,2.407 | 1.801** | 1.227,2.643 | | 1.882*** | 1.297,2.732 | |
| Urbanisation | 1.135 | 0.697,1.848 | 1.193 | 0.739,1.927 | | 1.162 | 0.726,1.858 | |
| Public healthcare spending | 1.009 | 1.000,1.019 | 1.007 | 0.998,1.017 | | 1.005 | 0.995,1.016 | |
| Public hospital beds | 1.001 | 0.941,1.065 | 0.99 | 0.934,1.048 | | 0.994 | 0.938,1.053 | |
| Private hospital beds | 1.193 | 0.913,1.561 | 1.231 | 0.940,1.614 | | 1.206 | 0.928,1.567 | |
| Private healthcare insurance | 0.831** | 0.744,0.928 | 0.857** | 0.772,0.952 | | 0.873* | 0.784,0.973 | |
| GDP | 0.846** | 0.759,0.944 | 0.880* | 0.794,0.976 | | 0.903* | 0.816,1.000 | |
| (Private healthcare insurance) x (GDP) | 0.953*** | 0.934,0.972 | 0.959*** | 0.941,0.977 | | 0.963*** | 0.945,0.982 | |
|  |  |  |  |  | |  |  | |
| N (Observations) | 22,384 |  | 22,384 |  | | 22,384 |  | |
| N (Municipalities) | 1,599 |  | 1,599 |  | | 1,599 |  | |

Exponentiated coefficients; * p<0.05, ** p<0.01, *** p<0.001 RR- Rate Ratio; 95% CI- 95% confidence interval; ESF - Estratégia de Saúde da Família (Family Health Strategy); GDP – Gross Domestic Product;

Notes: The study period was from 2000 to 2013. Robust standards errors employed. ESF coverage is a two year average of within year municipal ESF coverage and coverage in the year before. Year is a continuous variable and is interpreted as the underlying annual change in mortality rate during the study period. ESF coverage, poverty rate and the urbanisation rate are all expressed as percentages and scaled so a 1 unit increase represents a 100% increase. Bolsa Família coverage (cont) is also expressed as a percentage and scaled so a 1 unit increase represents a 100% increase, whereas Bolsa Família coverage (cat) is categorical variable. Private healthcare insurance is also expressed as a percentage, but is log transformed. Illiteracy is the illiteracy rate of those aged 25 and over and is log transformed. Public healthcare spending is expressed as R$100s per person as is GDP, although GDP is log transformed. Public and private hospital beds are expressed per 1,000 municipal inhabitants. Some municipalities and/or year observations not included due to no deaths from ambulatory care sensitive conditions for that racial group.

**Table B - Results from longitudinal fixed-effects Poisson regression of ACSC mortality in black/ *pardo* population with an interaction between ESF coverage and Bolsa Família coverage**

|  | **M1** | | **M2** | | **M3** | | |  |
| --- | --- | --- | --- | --- | --- | --- | --- | --- |
|  | **RR** | **95% CI** | **RR** | **95% CI** | | **RR** | **95% CI** | |
| ESF coverage | 0.921*** | 0.881,0.963 | 0.939** | 0.898,0.982 | | 0.931** | 0.889,0.975 | |
| Year | 0.972*** | 0.964,0.979 | 0.975*** | 0.967,0.983 | | 0.975*** | 0.967,0.983 | |
| Bolsa Família coverage (cont) | 0.826 | 0.682,1.001 | - | - | | - | - | |
| Bolsa Família coverage (cat) |  |  |  |  | |  |  | |
| None (0%) | - | - | 1.000 | Ref | | 1.000 | Ref | |
| Medium (0-20%) | - | - | 0.961*** | 0.947,0.976 | | 0.957*** | 0.939,0.976 | |
| High (20%+) | - | - | 0.969* | 0.944,0.995 | | 0.971 | 0.923,1.023 | |
| ESF * Bolsa Família coverage |  |  |  |  | |  |  | |
| ESF coverage * None (0%) | - | - | - | - | | 1.000 | Ref | |
| ESF coverage * Med (0-20%) | - | - | - | - | | 1.016 | 0.973,1.060 | |
| ESF coverage * High (20%+) | - | - | - | - | | 1.004 | 0.932,1.081 | |
| Illiteracy | 0.908 | 0.799,1.031 | 0.933 | 0.816,1.066 | | 0.935 | 0.818,1.069 | |
| Poverty | 1.172 | 0.864,1.590 | 1.303 | 0.970,1.751 | | 1.317 | 0.980,1.772 | |
| Urbanisation | 0.83 | 0.579,1.189 | 0.883 | 0.618,1.262 | | 0.881 | 0.617,1.258 | |
| Public healthcare spending | 1.002 | 0.991,1.012 | 1.001 | 0.990,1.012 | | 1.001 | 0.990,1.012 | |
| Public hospital beds | 1.012 | 0.959,1.068 | 1.003 | 0.952,1.057 | | 1.004 | 0.953,1.057 | |
| Private hospital beds | 1.097 | 0.927,1.296 | 1.134 | 0.968,1.328 | | 1.131 | 0.965,1.324 | |
| Private healthcare insurance | 0.906* | 0.834,0.985 | 0.915* | 0.844,0.993 | | 0.917* | 0.845,0.994 | |
| GDP | 0.861*** | 0.789,0.939 | 0.872** | 0.800,0.951 | | 0.874** | 0.802,0.953 | |
| (Private healthcare insurance) x (GDP) | 0.976** | 0.960,0.992 | 0.978** | 0.963,0.994 | | 0.978** | 0.963,0.994 | |
|  |  |  |  |  | |  |  | |
| N (Observations) | 22,694 |  | 22,694 |  | | 22,694 |  | |
| N (Municipalities) | 1,621 |  | 1,621 |  | | 1,621 |  | |

Exponentiated coefficients; * p<0.05, ** p<0.01, *** p<0.001 RR- Rate Ratio; 95% CI- 95% confidence interval; ESF - Estratégia de Saúde da Família (Family Health Strategy); GDP – Gross Domestic Product;

Notes: The study period was from 2000 to 2013. Robust standards errors employed. ESF coverage is a two year average of within year municipal ESF coverage and coverage in the year before. Year is a continuous variable and is interpreted as the underlying annual change in mortality rate during the study period. ESF coverage, poverty rate and the urbanisation rate are all expressed as percentages and scaled so a 1 unit increase represents a 100% increase. Bolsa Família coverage (cont) is also expressed as a percentage and scaled so a 1 unit increase represents a 100% increase, whereas Bolsa Família coverage (cat) is categorical variable. Private healthcare insurance is also expressed as a percentage, but is log transformed. Illiteracy is the illiteracy rate of those aged 25 and over and is log transformed. Public healthcare spending is expressed as R$100s per person as is GDP, although GDP is log transformed. Public and private hospital beds are expressed per 1,000 municipal inhabitants. Some municipalities and/or year observations not included due to no deaths from ambulatory care sensitive conditions for that racial group.

**Table C - Results from longitudinal fixed-effects Poisson regression of ACSC mortality in white population with an interaction between ESF coverage and Bolsa Família coverage**

The ESF *Bolsa Família interaction is non-significant overall at p=0.118.

|  | **M1** | | **M2** | | **M3** | | |
| --- | --- | --- | --- | --- | --- | --- | --- |
|  | **Coeff.** | **95% CI** | **Coeff.** | **95% CI** | **Coeff.** | **95% CI** | |
| ESF coverage | -0.179* | -0.336,-0.022 | -0.173* | -0.329,-0.016 | -0.236* | -0.419,-0.052 | |
| Year | 0.010 | -0.021,0.041 | 0.011 | -0.020,0.042 | 0.013 | -0.019,0.044 | |
| Bolsa Família coverage (cont) | -0.170 | -0.549,0.209 | - | - | - | - | |
| Bolsa Família coverage (cat) |  |  |  |  |  |  | |
| None (0%) | - | - | 1.000 | Ref | 1.000 | Ref | |
| Medium (0-20%) | - | - | -0.035 | -0.130,0.059 | -0.117 | -0.239,0.005 | |
| High (20%+) | - | - | -0.066 | -0.167,0.035 | -0.044 | -0.224,0.137 | |
| ESF * Bolsa Família coverage |  |  |  |  |  |  | |
| ESF coverage * None (0%) | - | - | - | - | 1.000 | Ref | |
| ESF coverage * Med (0-20%) | - | - | - | - | 0.164 | -0.001,0.330 | |
| ESF coverage * High (20%+) | - | - | - | - | 0.010 | -0.207,0.227 | |
| Illiteracy | 0.066 | -0.476,0.609 | 0.071 | -0.468,0.611 | 0.080 | -0.459,0.618 | |
| Poverty | 1.226* | 0.167,2.284 | 1.257* | 0.173,2.341 | 1.320* | 0.232,2.408 | |
| Urbanisation | 0.847 | -0.361,2.056 | 0.856 | -0.343,2.055 | 0.817 | -0.378,2.013 | |
| Public healthcare spending | 0.012 | -0.014,0.039 | 0.012 | -0.015,0.038 | 0.009 | -0.019,0.036 | |
| Public hospital beds | -0.082 | -0.217,0.054 | -0.086 | -0.222,0.050 | -0.088 | -0.223,0.047 | |
| Private hospital beds | 0.088 | -0.207,0.382 | 0.099 | -0.192,0.389 | 0.105 | -0.188,0.397 | |
| Private healthcare insurance | -0.114 | -0.405,0.177 | -0.101 | -0.381,0.180 | -0.100 | -0.380,0.181 | |
| GDP | -0.132 | -0.450,0.185 | -0.117 | -0.423,0.189 | -0.107 | -0.414,0.200 | |
| (Private healthcare insurance) x (GDP) | -0.038 | -0.095,0.019 | -0.035 | -0.090,0.020 | -0.0342 | -0.089,0.021 | |
|  |  |  |  |  |  |  | |
| N (Observations) | 21,336 |  | 21,336 |  | 21,336 |  | |
| N (Municipalities) | 1,622 |  | 1,622 |  | 1,622 |  | |
| * p<0.05, ** p<0.01, *** p<0.001 Coeff – Coefficient; 95% CI- 95% confidence interval; ESF - Estratégia de Saúde da Família (Family Health Strategy); GDP – Gross Domestic Product;  Notes: The study period was from 2000 to 2013. Robust standards errors employed. ESF coverage is a two year average of within year municipal ESF coverage and coverage in the year before. Year is a continuous variable and is interpreted as the underlying annual change in mortality rate during the study period. ESF coverage, poverty rate and the urbanisation rate are all expressed as percentages and scaled so a 1 unit increase represents a 100% increase. Bolsa Família coverage (cont) is also expressed as a percentage and scaled so a 1 unit increase represents a 100% increase, whereas Bolsa Família coverage (cat) is categorical variable. Private healthcare insurance is also expressed as a percentage, but is log transformed. Illiteracy is the illiteracy rate of those aged 25 and over and is log transformed. Public healthcare spending is expressed as R$100s per person as is GDP, although GDP is log transformed. Public and private hospital beds are expressed per 1,000 municipal inhabitants. Some municipalities and/or year observations not included due to no deaths from ambulatory care sensitive conditions for that racial group. | | | | | | |  |

**References**

1. Guanais FC. The combined effects of the expansion of primary health care and conditional cash transfers on infant mortality in Brazil, 1998-2010. Am J Public Health. 2013;103(11):2000-6.
