# Supplementary material for: Association between expansion of primary healthcare and racial inequalities in mortality amenable to primary care in Brazil: A national longitudinal analysis
Source: PLoS Med. 2017 May 30;14(5):e1002306. doi: 10.1371/journal.pmed.1002306 (PMC5448733; doi:10.1371/journal.pmed.1002306)
Supplement: S1 Table — (DOCX) [file pmed.1002306.s010.docx]

**S1 Table – National age-standardised mortality rates (deaths per 100,000) from ambulatory-care-sensitive conditions in black/*pardo* and white groups, absolute difference in rates, and standardised rate ratio in 1,622 municipalities with adequate reporting of vital statistics (2000–2013).**

|  | **2000** | **2001** | **2002** | **2003** | **2004** | **2005** | **2006** | **2007** | **2008** | **2009** | **2010** | **2011** | **2012** | **2013** |
| --- | --- | --- | --- | --- | --- | --- | --- | --- | --- | --- | --- | --- | --- | --- |
| White | 75.6 | 71.9 | 68.7 | 66.4 | 64.3 | 59.7 | 59.5 | 58.6 | 56.7 | 54.8 | 54.0 | 53.9 | 50.4 | 49.2 |
| Black/*Pardo* | 93.3 | 86.1 | 80.0 | 78.4 | 77.0 | 70.0 | 70.3 | 68.8 | 67.6 | 65.3 | 65.0 | 63.9 | 60.9 | 58.0 |
|  |  |  |  |  |  |  |  |  |  |  |  |  |  |  |
| Difference | 17.7 | 14.2 | 11.3 | 12.0 | 12.7 | 10.4 | 10.8 | 10.1 | 11.2 | 10.5 | 11.0 | 10.0 | 10.6 | 8.7 |
| SRR | 1.23 | 1.20 | 1.16 | 1.18 | 1.20 | 1.17 | 1.18 | 1.17 | 1.20 | 1.19 | 1.20 | 1.19 | 1.21 | 1.18 |

SRR – Standardised Rate Ratio

Notes: The age-standardised mortality rates are of those aged 70 years and younger. The difference between the mortality rates is the absolute difference between the black /*pardo* group and the white group. The SRR is the ratio between the black /*pardo* group and the white group.
