# Supplementary material for: Association between expansion of primary healthcare and racial inequalities in mortality amenable to primary care in Brazil: A national longitudinal analysis
Source: PLoS Med. 2017 May 30;14(5):e1002306. doi: 10.1371/journal.pmed.1002306 (PMC5448733; doi:10.1371/journal.pmed.1002306)
Supplement: S2 Table — (DOCX) [file pmed.1002306.s011.docx]

**S2 Table – Sensitivity analysis: different classifications of Estratégia de Saúde da Família coverage**

Two-year average ESF (*Estratégia de Saúde da Família*) coverage was employed in the main analysis to account for lagged and duration effects of the ESF over two years, as well as interpretability. Below are the results showing alternative classifications of ESF coverage. Covariates and model specifications are identical (except ESF coverage) to the main analyses. Model 1 shows ESF classification as a two-year average (used in the main analyses). Model 2 shows the associations using only within-year ESF coverage, whereas Model 3 shows ESF coverage including both within-year ESF coverage and ESF coverage in the year prior.

**Results from longitudinal fixed-effects Poisson regression of ACSC mortality in black/ *pardo* and white populations with different classifications of ESF** **coverage**

|  |  | | | |  |  |  | |  |  |
| --- | --- | --- | --- | --- | --- | --- | --- | --- | --- | --- |
|  | **Model 1** |  | | **Model 2** | |  | **Model 3** |  | | |
|  | **RR** | **95% CI** | | **RR** | | **95% CI** | **RR** | **95% CI** | | |
| ***Black / Pardo*** |  |  | |  | |  |  |  | | |
| Two-year average ESF coverage | 0.846*** | 0.796,0.899 | | - | | - | - | - | | |
| ESF coverage (within year) | - | - | | 0.877*** | | 0.831,0.926 | 0.940* | 0.891,0.990 | | |
| ESF coverage (year before) | - | - | | - | | - | 0.902*** | 0.856,0.950 | | |
| N (Observations) | 22,384 |  | | 22,384 | |  | 22,384 |  | | |
|  |  |  | |  | |  |  |  | | |
| ***White*** |  |  | |  | |  |  |  | | |
| Two-year average ESF coverage | 0.932** | 0.892,0.974 | | - | | - | - | - | | |
| ESF coverage (within year) | - | - | | 0.933*** | | 0.898,0.970 | 0.939** | 0.903,0.977 | | |
| ESF coverage (year before) | - | - | | - | | - | 0.991 | 0.954,1.029 | | |
| N (Observations) | 22,694 |  | | 22,694 | |  | 22,694 |  | | |
|  |  | |  | |  |  |  | |  |  |
| Exponentiated coefficients; * p<0.05, ** p<0.01, *** p<0.001 RR- Rate Ratio; 95% CI- 95% confidence interval; ESF - Estratégia de Saúde da Família (Family Health Strategy); GDP – Gross Domestic Product;  Notes: The study period was from 2000 to 2013. Robust standards errors employed. Year is a continuous variable and is interpreted as the underlying annual change in mortality rate during the study period. All measures of ESF coverage are expressed as percentages and scaled so a 1 unit increase represents a 100% increase Although not shown, all regressions controlled for: Bolsa *Família* coverage (%); Illiteracy rate of those over 25 (Log); Poverty rate (%); Urban rate (%); Public Healthcare spending (R$100 per person); Public hospital beds per 1,000 population; Private hospital beds per 1,000 population; Private healthcare insurance (%) (Log); GDP per person (R$100) (Log); Private healthcare insurance (%) (Log)* GDP per person (R$100) (Log) Interaction. Some municipalities and/or year observations not included due to no deaths from ambulatory care sensitive conditions for that racial group. | | | | | | | | | | |
