# Supplementary material for: Association between expansion of primary healthcare and racial inequalities in mortality amenable to primary care in Brazil: A national longitudinal analysis
Source: PLoS Med. 2017 May 30;14(5):e1002306. doi: 10.1371/journal.pmed.1002306 (PMC5448733; doi:10.1371/journal.pmed.1002306)
Supplement: S4 Table — (DOCX) [file pmed.1002306.s013.docx]

**S4 Table – Comparison of ambulatory-care-sensitive conditions and mortality amenable to primary care.**

ACSCs were the main outcome of the analysis. ACSCs have been infrequently used to study mortality, often being used for hospitalisations [[1-3](#_ENREF_1)]. More commonly, amenable mortality has been examined in health services research [[4](#_ENREF_4),[5](#_ENREF_5)], but this is not specific to primary care. Below is a comparison of the ACSC list and a list of amenable mortality by Nolte and Mckee (2008) [[4](#_ENREF_4)].

| **Condition** | **ICD-10 Codes** | **ACSC*** | **Amenable** | **Amenable ages** |
| --- | --- | --- | --- | --- |
| *Vaccine preventable diseases* |  |  |  |  |
| Tetanus neonatorum | A33 | Y | N |  |
| Obstetrical tetanus | A34 | Y | Y | 0-74 years |
| Other tetanus | A35 | Y | Y | 0-74 years |
| Diphtheria | A36 | Y | Y | 0-74 years |
| Whooping cough | A37 | Y | Y | 0-14 years |
| Acute poliomyelitis | A80 | N | Y | 0-74 years |
| Yellow fever | A95 | Y | N |  |
| Acute hepatitis B | B16 | Y | N |  |
| Measles | B05 | Y | Y | 1-14 years |
| Rubella | B06 | Y | N |  |
| Mumps | B26 | Y | N |  |
| Sequelae of tuberculosis | B90 | N | Y | 0-14 years |
| Haemophilus meningitis | G00.0 | Y | N |  |
| Tuberculous meningitis | A17.0 | Y | Y | 0-74 years |
| Miliary tuberculosis | A19 | Y | Y | 0-74 years |
|  |  |  |  |  |
| *Preventable conditions* |  |  |  |  |
| Tuberculosis | A15-A16, A17.1-A17.9, A18 | Y | Y | 0-74 years |
| Acute rheumatic fever | I00-I02 | Y | N |  |
| Syphilis (Early and Late) | A51-A53 | Y | N |  |
| Malaria | B50-B54 | Y | N |  |
| Ascariasis | B77 | Y | N |  |
|  |  |  |  |  |
| *Gastrointestinal infections and complications* |  |  |  |  |
| Intestinal infectious diseases | A00-A09 | Y | Y | 0-14 years |
| Dehydration | E86 | Y | N |  |
|  |  |  |  |  |
| *Anaemia* | D50 | Y | N |  |
|  |  |  |  |  |
| *Cancers* |  |  |  |  |
| Malignant neoplasm of colon and rectum | C18-21 | N | Y | 0-74 years |
| Malignant neoplasm of skin | C44 | N | Y | 0-74 years |
| Malignant neoplasm of breast | C50 | N | Y | 0-74 years |
| Malignant neoplasm of cervix uteri | C53 | N | Y | 0-74 years |
| Malignant neoplasm of cervix uteri and uterus | C54, C55 | N | Y | 0-44 years |
| Malignant neoplasm of testis | C62 | N | Y | 0-74 years |
| Hodgkin’s disease | C81 | N | Y | 0-74 years |
| Leukaemia | C91-5 | N | Y | 0-44 years |
|  |  |  |  |  |
| *Nutritional deficiencies* |  |  |  |  |
| Malnutrition | E40-E46 | Y | N |  |
| Other nutritional deficiencies | E50-E64 | Y | N |  |
|  |  |  |  |  |
| *Infections of the ear, nose and throat* |  |  |  |  |
| Otitis media | H66 | Y | N |  |
| Acute upper respiratory infections | J00-J03, J06, J31, J45-J46 | Y | Y | 1-14 years |
| Acute obstructive laryngitis, zoonotic influenza | J05, J09 | N | Y | 1-14 years |
| Influenza | J10 J11 | N | Y | 0-74 years |
| Pneumonia | J12 | N | Y | 0-74 years |
|  |  |  |  |  |
| *Bacterial pneumonias* |  |  |  |  |
| Bacterial pneumonias | J13-J14 | Y | Y | 0-74 years |
| Selected infectious pneumonias | J15.1-J15.2, J15.5-J15.7, J16, J17, J18.0 J18.2-J18.9 | N | Y | 0-74 years |
| Selected infectious pneumonias | J15.3-J15.4, J15.8-J15.9, J18.1 | Y | Y | 0-74 years |
| *Other diseases of the respiratory tract* |  |  |  |  |
| Bronchitis, asthma COPD, bronchiectasis | J20, J21, J40-J47 | Y | Y | 1-14 years |
| Other respiratory diseases | J22-J39, J48-J99 | N | Y | 1-14 years |
|  |  |  |  |  |
| *Cardiovascular disease* |  |  |  |  |
| Chronic rheumatic heart disease | I05-I09 | N | Y | 0-74 years |
| Hypertension | I10-I11 | Y | Y | 0-74 years |
| Hypertensive renal disease and secondary hypertension | I12-I13, I15 | N | Y | 0-74 years |
| Angina | I20 | Y | Y (only 50%) | 0-74 years |
| Myocardial infarction and ischaemic heart diseases | I21-I25 | N | Y (only 50%) | 0-74 years |
| Heart failure | I50, J81 | Y | N |  |
| Brain haemorrhage | I60-I62, I68 | N | Y | 0-74 years |
|  |  |  |  |  |
| *Cerebrovascular disease* |  |  |  |  |
| Cerebral infarction, stroke, | I63-I67, I69 | Y | Y | 0-74 years |
| Transient cerebral ischaemic attacks | G45-G46 | Y | N |  |
| Thyroid disease | E00-E07 | N | Y | 0-74 years |
|  |  |  |  |  |
| *Diabetes mellitus* | E10-E14 | Y | Y | 0-49 years |
|  |  |  |  |  |
| *Epilepsy* | G40-G41 | Y | Y | 0-74 years |
|  |  |  |  |  |
| *Infections of the kidney and urinary tract* |  |  |  |  |
| Nephritis and nephrosis | N00-N07, N17-N19, N25-N27 | N | Y | 0-74 years |
| Nephritis | N10-N12 | Y | N |  |
| Cystitis | N30 | Y | N |  |
| Urethritis and urethral syndrome | N34 | Y | N |  |
| Urinary tract infection | N39.0 | Y | N |  |
| Benign prostatic hyperplasia | N40 | N | Y | 0-74 years |
|  |  |  |  |  |
| *Infections of the skin and subcutaneous tissue* | A46, L01-L04, L08 | Y | N |  |
|  |  |  |  |  |
| *Pelvic inflammatory disease* | N70-N73, N75-N76 | Y | N |  |
|  |  |  |  |  |
| *Gastric ulcers* |  |  |  |  |
| Peptic ulcer | K25-K27 | Y | Y | 0-74 years |
| Peptic ulcer | K28 | Y | N |  |
| Appendicitis | K35-K38 | N | Y | 0-74 years |
| Abdominal hernia | K40-K46 | N | Y | 0-74 years |
| Cholelithiasis and cholecystitis | K80-K81 | N | Y | 0-74 years |
| Haematemesis, melaena, gastric haemorrhage | K92.0-K92.2 | Y | N |  |
|  |  |  |  |  |
| *Diseases of the prenatal period and childbirth* |  |  |  |  |
| Urinary tract infection during pregnancy | O23 | Y | Y | 0-74 years |
| All other causes of maternal death | O00-O22, O24-O99 | N | Y | 0-74 years |
| Congenital syphilis | A50 | Y | N |  |
| Congenital rubella | P35.0 | Y | Y | 0-74 years |
| All other causes of perinatal death | P00-P34, P35.1-P96 | N | Y | 0-74 years |
| Congenital cardiovascular anomalies | Q20-Q28 | N | Y | 0-74 years |
| Misadventures to patients during surgical and medical care | Y60-9, Y83-4 | N | Y | 0-74 years |
|  |  |  |  |  |

* In this study, all deaths from ACSCs under the age of 70 years were included in this analysis.

Source: Alfradique ME et al, (2009) and Nolte and McKee (2008).

**References**

1. Guanais F, Macinko J. Primary care and avoidable hospitalizations: evidence from Brazil. The Journal of ambulatory care management. 2009;32(2):115-22.

2. Macinko J, de Oliveira VB, Turci MA, Guanais FC, Bonolo PF, Lima-Costa MF. The influence of primary care and hospital supply on ambulatory care-sensitive hospitalizations among adults in Brazil, 1999-2007. Am J Public Health. 2011;101(10):1963-70.

3. Macinko J, Dourado I, Aquino R, Bonolo Pde F, Lima-Costa MF, Medina MG, et al. Major expansion of primary care in Brazil linked to decline in unnecessary hospitalization. Health Aff (Millwood). 2010;29(12):2149-60. Epub 2010/12/08. doi: 10.1377/hlthaff.2010.0251. PubMed PMID: 21134915.

4. Nolte E, McKee CM. Measuring the health of nations: updating an earlier analysis. Health Aff (Millwood). 2008;27(1):58-71. doi: 10.1377/hlthaff.27.1.58. PubMed PMID: 18180480.

5. Hone T, Rasella D, Barreto M, Atun R, Majeed A, Millett C. Large Reductions In Amenable Mortality Associated With Brazil’s Primary Care Expansion And Strong Health Governance. Health Aff (Millwood). 2017;36(1):149-58.
