# Supplementary material for: Association between expansion of primary healthcare and racial inequalities in mortality amenable to primary care in Brazil: A national longitudinal analysis
Source: PLoS Med. 2017 May 30;14(5):e1002306. doi: 10.1371/journal.pmed.1002306 (PMC5448733; doi:10.1371/journal.pmed.1002306)
Supplement: S1 Text — (DOCX) [file pmed.1002306.s014.docx]

**S1 Text – Methods for redistribution of ill-defined deaths**

The reductions in reporting of ill-defined causes over time can generate artificial trends. The number of defined deaths will show a concomitant increase as ill-defined deaths fall over time, resulting in bias especially in fixed-effects longitudinal regression.

We use a recently published methodology by França et al (2014) to redistribute ill-defined deaths to defined causes [1]. This is based on death certificates which under-go investigation to check accuracy. The cause of death on some certificates is recoded to a different cause of death following investigation. By using those deaths originally from ill-defined causes who are reassigned to defined causes, we can calculate “re-distribution rates” for different causes. This offers a superior methodology to traditional methods – where ill-defined causes are reassigned based on the distribution of defined causes – as deaths originally defined as ill-defined causes are likely to be from different causes [1].

We calculate redistribution rates by for overall ACSC mortality and by groups of ACSC (for each age group and state) for the period of 2007-2012 (when death certificate investigation took place). Where states did not investigate deaths, national average redistribution rates were used. Averages for 2007-2012 were applied to the whole study period as time trends were not evident. Using the redistribution rates for ACSCs and groups of ACSCs, we re-classified ill-defined deaths and calculated an adjusted number of deaths.

An adjusted number of deaths was calculated based on:

$${\begin{matrix} \mathrm{Adjusted} \\ \mathrm{deaths} \end{matrix}}_{i,j,k,x}= \begin{matrix} \mathrm{reported} \\ \mathrm{deaths}_{i,j,k,x} \end{matrix}+ \left( \begin{matrix} ill-defined \\ \mathrm{deaths}_{i,j,k} \end{matrix}*\begin{matrix} re-distribution \\ \mathrm{rate}_{i,j,x} \end{matrix} \right)$$

Where$i=$municipality, $j=$age group, $k=$ year, and $x$ = defined causes (from ACSCs).

**References**

1. França E, Teixeira R, Ishitani L, Duncan BB, Cortez-Escalante JJ, Morais Neto OLd, et al. Ill-defined causes of death in Brazil: a redistribution method based on the investigation of such causes. Revista de saude publica. 2014;48(4):671-81.
