# Supplementary material for: Association between expansion of primary healthcare and racial inequalities in mortality amenable to primary care in Brazil: A national longitudinal analysis
Source: PLoS Med. 2017 May 30;14(5):e1002306. doi: 10.1371/journal.pmed.1002306 (PMC5448733; doi:10.1371/journal.pmed.1002306)
Supplement: S2 Text — (DOCX) [file pmed.1002306.s015.docx]

**S2 Text – Methods for imputing missing race**

Over the period 2000-2013, race was missing on 5.84% of death certificates in municipalities with adequate reporting of vital statistics. Missing values for race were imputed using other variables available on the death certificate including age, sex, education status, marital status, location of death, if the death was attended by a doctor and if the death was due to violent causes. Additionally, the percentage of the population that was either white or black in the municipality for each year (and each specific five-year age group) was used as a predictor of race.

A probit model was undertaken – suitable for multiple categorical outcomes – predicting either white, black/*pardo* or other (including indigenous, or amarello) as the outcome. The true positive rate of the prediction was calculated based on all death certificates including death certificates with known race showing a 68.0% true positive rate.
