# Supplementary material for: Association between expansion of primary healthcare and racial inequalities in mortality amenable to primary care in Brazil: A national longitudinal analysis
Source: PLoS Med. 2017 May 30;14(5):e1002306. doi: 10.1371/journal.pmed.1002306 (PMC5448733; doi:10.1371/journal.pmed.1002306)
Supplement: S3 Text — (DOCX) [file pmed.1002306.s016.docx]

**S3 Text - Fixed-effect longitudinal Poisson regression model specification**

**Longitudinal models**

Longitudinal models, also called panel models, are used when the unit of analysis has repeated observations over time [1]. Data points of each unit of analysis over time are likely to be highly correlated (i.e. they are the same unit of analysis) and so analyses must take the clustered nature of the data into account [1]. In this study, municipalities have annual data permitting longitudinal data analysis.

**Fixed and random effects**

There is considerable flexibility in analysing longitudinal datasets depending on assumptions made – including whether to employ random or fixed-effects. Within the econometric literature, fixed- and random-effects refer to assumptions about the associations of error terms within the model[1].

The general model [1, 2] for both fixed and random-effects is specified as:

$\text{y}_{\text{it}}\text{=}{\text{ }\text{x}}_{\text{it}}\text{β +}{\text{ }\text{ α}_{\text{i}}\text{ + ε}}_{\text{it}}$

Where *y_it_* is the dependent variable for unit *i* at time *t*, and *x_it_* is the independent variable of unit *i* at time *t* with coefficient β. Both are error terms - with *α_i_* the random individual-specific effects (time invariant) for unit *i*, and *ε_it_* the idiosyncratic error (time variant) that related to an individual unit *i* at time *t*.

In random-effect models, it is assumed that *α_i_* is uncorrelated with any independent variables *x_it_* at any time. In other words, unobserved effects are only randomly associated with explanatory variables in the model [1]. This is a strong assumption to make and is likely to be violated – especially in models where only limited explanatory variables are available [2].

In fixed-effect models, *α_i_* is permitted to correlate with independent variables *x_it_* (i.e. unobserved characteristics can be associated with explanatory variables), which is a less stringent assumption to make. Fixed-effect models have the ability to control for these constant (or stable) unobserved characteristics over time and estimates unbiased of any correlated between errors and explanatory variables are obtained. Additionally, the choice of fixed- or random-effects can be based on the Hausman specification test, which compares error terms between the models.

**Poisson model**

A Poisson distribution is used when the outcomes are count variables – e.g. the number of deaths – taking non-negative integer values (e.g. 0, 1, 2, etc) over a fixed time period – e.g. a year [3]. The Poisson model is also appropriate for use in longitudinal data models [3] and is estimated by log function to ensure positive values [2]. Furthermore, the Poisson model can be re-parameterised as a rate model using an exposure or offset to account for the time or area that events can occur in [3]. This is important for this study as populations vary by municipalities in Brazil and thus the number of deaths will vary by population size and mortality rate. Using an exposure or offset (the annual population) can take this into account (demonstrated below).

One characteristic of the Poisson model is that a distribution’s mean is equal to its variance, but this is often violated. Indeed, the distribution of mortality rates in Brazil is over-dispersed. Negative Binomial regressions are often used to deal with the violation of this assumption, but there are problems with these models also. It has been shown by Allison and Waterman (2002) [4], that negative binomial models in common statistical software may in fact not be true fixed effects allowing estimation of time-invariant characteristics. Because of these concerns, and problems fitting fixed-effect negative binomial models to the data, we use a Poisson distribution for analysis. Highly comparable results are obtained using negative binomial models.

**Fixed-effects longitudinal Poisson regression**

The regression model used in the equation can be shown as:

*log*(*y_it_) = α_i_ + β_1_ x_1 it_ + β_k_ x_k it_ + β_2_ t + ε_it_ + log(pop_it_)*

Where:

*t* refers to the year (from 2000 to 2012) and *i* refers to an individual municipality;

*y_it_* is the number of deaths from ACSCs in municipality *i* in year *t*;

*x_1 it_* is the coverage (from 0 to 100%) of ESF in municipality *i* in year *t* with a coefficient of *β_1_;*

*x_k it_* refers to each covariate (*k)* for municipality *i* in year *t* with coefficient *β_k_;*

*β_2_ t* is the yearly trend estimated with coefficient *β_2_;*

*α_i_* is the fixed effect (municipality-level time-invariant effect) error term (not estimated);

*ε_it_* is the idiosyncratic error term for municipality *i* in year *t* (not estimated);

*pop_it_* is the population under 75 years of age in municipality *i* in year *t* (and acts as an offset term);

The offset term allows the independent variable (deaths from ACSCs) to be expressed per population:

*log*(*y_it_) - log(pop_it_)= α_i_ + β_1_ x_1 it_ + β_k_ x_k it_ + β_2_ t + ε_it_*

*log*(*y_it_ / pop_it_)= α_i_ + β_1_ x_1 it_ + β_k_ x_k it_ + β_2_ t + ε_it_*

The parameters are often exponentiated to aid interpretation:

*y_it_ / pop_it_= exp( α_i_ + β_1_ x_1 it_ + β_k_ x_k it_ + β_2_ t + ε_it_ )*

The exponentiated coefficients of traditional (non-offset) Poisson models are interpreted as incident rate ratios. Using an off-set model, the we obtain a rate ratio (RR). For example, the exponentiated coefficient *β_1_* is the estimated change in the ACSC mortality rate from a 100% expansion of ESF and is our main variable of interest in the analysis. A value of 0.92 refers to a ratio between the mortality rates of 100% (*x_it_* = 1) and 0% (*x_it_* = 0) ESF coverage, and is thus interpreted as an associated 8% reduction in ACSC mortality. Because this is a fixed-effect longitudinal regression, we only associate this to changes *within* the municipality (i.e. changes in *within-*municipality mortality and *within*-municipality ESF coverage). *Between*-municipality effects (i.e. difference between municipalities) are not estimated. For clarity, RR are reported in tables, and associated changes in mortality are reported in the text.

**References**

1. Cameron C, Triveda P. Microeconomics Using Stata, Revised Edition. Texas, USA: Stata Press; 2010. 706 p.

2. Wooldridge J. Introductory econometrics: A modern approach: Cengage Learning; 2012.

3. Hilbe J. Negative Binomial Regression. Cambridge, UK: Cambridge University Press; 2011.

4. Allison PD, Waterman RP. Fixed–effects negative binomial regression models. Sociological methodology. 2002;32(1):247-65.
