# Supplementary material for: Association between expansion of primary healthcare and racial inequalities in mortality amenable to primary care in Brazil: A national longitudinal analysis
Source: PLoS Med. 2017 May 30;14(5):e1002306. doi: 10.1371/journal.pmed.1002306 (PMC5448733; doi:10.1371/journal.pmed.1002306)
Supplement: S4 Text — (DOCX) [file pmed.1002306.s017.docx]

**S4 Text - Calculating statistical difference in coefficients between two different models**

The results from the two longitudinal regression models on black/*pardo* and white ACSC mortality were compared, and the coefficients from ESF coverage compared statistically. From the main regression results, ESF expansion (from 0 to 100%) was associated with reductions of 15.4% (RR: 0.846; 95% CI: 0.796-0.899) and 6.8% (RR: 0.932; 95% CI: 0.892-0.974) in black/*pardo* and white ACSC mortality respectively. Whilst the confidence intervals overlap, the difference can still be significantly different. The Z test statistics for comparing the two coefficients was calculated using the formula [1]:

$$Z= \frac{\beta_{1}- \beta_{2}}{\sqrt{{(SE\beta_{1})}^{2}+{(SE\beta_{2})}^{2}}}$$

Where $\beta$ are the coefficients and$SE\beta$ are the standard errors from the two regressions. The Z-test statistics were converted to p-values for reporting.

Where the difference between two coefficients has been calculated, the p-value is reported in the text.

**References**

1. Clogg CC, Petkova E, Haritou A. Statistical methods for comparing regression coefficients between models. American Journal of Sociology. 1995:1261-93.
